# Supplementary material for: Defect-induced helicity dependent terahertz emission in Dirac semimetal PtTe2 thin films
Source: Nat Commun. 2024 Mar 23;15:2605. doi: 10.1038/s41467-024-46821-8 (PMC10960839; doi:10.1038/s41467-024-46821-8)
Supplement: Supplementary file 1 — Supplementary Information [file 41467_2024_46821_MOESM1_ESM.pdf]

Supplementary Information

Defect-induced helicity dependent terahertz emission in Dirac semimetal $PtTe_2$ thin films

Zhongqiang Chen[1,†], Hongsong Qiu[2,†], Xinjuan Cheng[3,†], Jizhe Cui[4], Zuanming Jin[5], Da Tian[2], Xu Zhang[1], Kankan Xu[1], Ruxin Liu[1], Wei Niu[1], Liqi Zhou[6], Tianyu Qiu[7], Yequan Chen[1], Caihong Zhang[2], Xiaoxiang Xi[7], Fengqi Song[7], Rong Yu[4], Xuechao Zhai[3,*], Biaobing Jin[2,8,*], Rong Zhang[1,9,*] & Xuefeng Wang[1,*]

[1]Jiangsu Provincial Key Laboratory of Advanced Photonic and Electronic Materials, State Key Laboratory of Spintronics Devices and Technologies, School of Electronic Science and Engineering, Collaborative Innovation Center of Advanced Microstructures, Nanjing University, Nanjing 210093, China

[2]Research Institute of Superconductor Electronics, School of Electronic Science and Engineering, MOE Key Laboratory of Optoelectronic Devices and Systems with Extreme Performances, Nanjing University, Nanjing 210093, China

[3]Department of Applied Physics, MIIT Key Laboratory of Semiconductor Microstructures and Quantum Sensing, Nanjing University of Science and Technology, Nanjing 210094, China

[4]School of Materials Science and Engineering, Tsinghua University, Beijing 100084, China

[5]Terahertz Technology Innovation Research Institute, Terahertz Spectrum and Imaging Technology Cooperative Innovation Center, University of Shanghai for Science and Technology, Shanghai 200093, China

[6]College of Engineering and Applied Sciences, Nanjing University, Nanjing 210093, China

[7]State Key Laboratory of Solid State Microstructures, School of Physics, Nanjing University, Nanjing 210093, China

[8]Purple Mountain Laboratories, Nanjing 211111, China

[9]Department of Physics, Xiamen University, Xiamen 361005, China

[†]These authors contributed equally to this work.

[*]**e-mail:** xfwang@nju.edu.cn; zhaixuechao@njust.edu.cn; bbjin@nju.edu.cn; rzhang@nju.edu.cn.

This PDF file includes:

Supplementary Text (Notes 1-6)

Supplementary Figures 1-27

Supplementary Tables 1-4

Supplementary References

Supplementary Text

Supplementary Note 1: Growth process and out-of-plane defect-gradient formation in $PtTe_2$ films

The Te source, reaction temperature and time, and the flow rate of carrier gas are all important factors for the growth of defect-gradient $PtTe_2$ films. The two-step growth process is schematically shown in Supplementary Fig. 1. Pt seeds with controlled thicknesses (1-10 nm) were firstly deposited on sapphire (Al_2O_3) substrates by magnetron sputtering at a fixed rate of 1.25 Å s^{-1}. Then the Pt-deposited Al_2O_3 substrate and Te powders were placed in a 10 mm-diameter quartz tube with the distance of ~5 cm. Notably, the used double-open quartz tube could accelerate the flow of Te vapor and allow more V_{Te} and a defect gradient to be formed in as-grown samples, which is very different from growth conditions in the previous reports.[1-3] Subsequently, the quartz tube was placed in a low-pressure CVD system with a tube diameter of 30 mm. After the CVD system was purged down to a base pressure of ~1 mTorr by the forming gas (95% Ar and 5% H_2), the furnace was ramped up to the temperature of 400 °C at a rate of 13.3 °C min^{-1}. During the growth process, the flow rate of forming gas was kept ~100 SCCM at a pressure of ~80 mTorr.

Next, we will introduce a detailed mechanism for the generation of a vertical defect gradient. The investigation of V_{Te} has attracted much attention in the $PtTe_2$ system, which is mainly focused on the sample surface.[4,5] However, the depth-profile defects remain largely unexplored yet. The formation of $PtTe_2$ films is primarily governed by two key steps (see Supplementary Fig. 2a): (i) the diffusion of Te atoms into a dense mass of Pt with a finite Te concentration gradient; (ii) the reaction between Pt and Te to yield $PtTe_2$. As the Pt content increases along the depth orientation, a diffusion barrier is formed along the van der Waals gap, implying the dominant diffusion-limited growth mechanism of $PtTe_2$.[6] The diffusion of Te into Pt can be quantitatively described by Fick's second law. The theoretical predictions and their validation can be described by:

$$\frac{\partial C}{\partial t} = \frac{\partial (D\frac{\partial C}{\partial x})}{\partial x} = D\frac{\partial^2 C}{\partial x^2} \tag{1}$$

where C is the concentration of the diffusion atoms, t is time, x is the diffusion length, and D is the diffusion coefficient. From the cross-sectional view of the STEM-HAADF images in Supplementary Fig. 8, it is found that the tellurization process occurs even in the lowest Pt layers close to the substrate. Hence, the V_{Te} formation arises from the re-evaporation and migration of Te atoms in as-grown $PtTe_2$ films, especially in a Te-poor environment. A V_{Te} defect gradient is produced in the $PtTe_2$ films with different thicknesses, which is attributed to its lower V_{Te} formation energy in the midst as compared to those at the top and bottom (see Supplementary Fig. 2b-d). This is almost consistent with our ptychographic reconstruction results shown in Fig. 1f. Actually, the choices of the substrate, the reaction temperature and time, and the flow rate of carrier gas should all play a critical role in the vertical defect distribution

in as-grown $PtTe_2$ films, which makes the statistical layer-dependent V_{Te} distribution (Fig. 1f) have the relatively large scatter and be slightly different from our theoretical prediction (Supplementary Fig. 2b-d). In brief, the V_{Te} concentration first increases and then decreases from the bottom to the top surface. Indeed, the systematic trend of a defect gradient really exists and the V_{Te} defect is mainly concentrated in the midst of films (Fig. 1f and Supplementary Fig. 2).

Moreover, note that if out-of-plane V_{Te} defect was randomly distributed, the band spin splitting and the inversion symmetry breaking would not be induced (Supplementary Fig. 24), which would contradict with our experimental observation of the second-order nonlinear photocurrents and the band structure calculations. Although the V_{Te} concentration is not so monotonically varied as expected, the out-of-plane V_{Te} defect gradient does form in the films and indeed plays a crucial role in the inversion symmetry breaking and the THz radiation.

Supplementary Note 2: Structural characterization by micro-Raman spectroscopy

Temperature-dependent Raman spectroscopic measurements have been well established to study the effect of defect states on the intrinsic phonon properties.[7] The room-temperature Raman spectra of as-grown $PtTe_2$ films show the typical E_g and A_{1g} modes at about 111.4 and 157.6 cm^{-1}, which corresponds to the in-plane and out-of-plane vibration of Te atoms, respectively (Supplementary Fig. 4b and 5). There are largely no noticeable peak shifts of the E_g mode for the as-grown and Te-passivated $PtTe_2$ films between 150 and 300 K (Supplementary Fig. 5c). As the temperature decreases, the blue shifts of both E_g and A_{1g} modes of the as-grown and Te-passivated $PtTe_2$ films can be seen from Supplementary Fig. 5, which can be attributed to the phonon-phonon interactions at low temperatures. This is similar to the case of other layered materials (e.g., MoS_2)[8] with the analogous structures. In addition, the larger blue shifts of two vibrational peaks at 5 K for the as-grown sample as compared to those for the Te-passivated sample can be attributed to the presence of the V_{Te} defect (Supplementary Fig. 5). The extracted temperature-dependent Raman peak positions of E_g and A_{1g} modes are shown in Supplementary Fig. 5c,d, respectively. As the temperature is no higher than 150 K, the difference of the E_g peak between two samples becomes larger, indicating that the contribution of the V_{Te} defect becomes more pronounced at low temperatures.

Considering the presence of the V_{Te} defect that affects the structural properties, next we carry out micro-Raman mapping at room temperature on a sub-millimeter scale to probe the spatial homogeneity of the defect-gradient $PtTe_2$ films. The corresponding Raman mapping images of the E_g and A_{1g} modes of $PtTe_2$ films clearly demonstrate the highly homogeneous colour contrasts from two arbitrary regions (with an interval of about 1 mm) on the single film (see Supplementary Fig. 6). Such spatially resolved Raman mapping images reveal the in-plane uniformity of the defect distribution on a millimeter scale of the as-grown $PtTe_2$ films. The in-plane uniformity can be further evidenced from the SHG mapping (Supplementary Fig. 22)

and laser-spot-dependent THz emission (Supplementary Fig. 15) on the single film, respectively, consistently ruling out the possible presence of the in-plane V_{Te} defect gradient.

Supplementary Note 3: Other mechanisms for the generation of the THz emission

First, the optical rectification process takes place upon the incident photon energy below the bandgap of semiconductors, which is the principal contribution to the THz emission in semiconductors.[9] Second, photocurrent effects such as the photo-Dember effect and the photothermal effect can be also responsible for the THz emission. Because the THz signal in our experiments is highly sensitive to the polarization state of the pump laser (Fig. 2), the above-mentioned two origins can be ruled out. Third, the photon drag effect (PDE) is also a classical effect for the polarization-dependent THz emission. The momentum of a single photon is small in the optical regime. However, the intense light beams with high photon flux can lead to the considerable currents.[10] The generation of PDE photocurrents requires photons with an oblique incidence to have a finite in-plane momentum that can be transferred to electrons along the momentum transfer direction to generate currents.[11,12] In view of the pump laser under normal incidence in our measurements, the PDE photocurrents are therefore almost negligible. In addition, the incidence-angle (α) dependent photocurrent polarity is absent, and the THz emission amplitude becomes noticeably smaller than that under normal incidence when α exceeds a value (i.e., >15°), further ruling out the PDE process (Supplementary Fig. 19). Finally, spin-galvanic effect (SGE) could be also a possible mechanism for the helicity dependent photocurrent. This effect was proposed in quantum well structures driven by electron spin.[13] The helicity dependent photocurrent can be generated by asymmetric spin-flip relaxation of spin-polarized electrons. However, the current polarity highly depends on the direction of the excited spins, as determined by the radiation helicity and the magnetic field. However, in our experiment, the polarity of the THz signals can be changed by the helicity of the laser without a magnetic field, thus ruling out the SGE.

From the above discussion of other possible mechanisms responsible for generating the THz emission, we corroborate that the photogalvanic effect is the dominant mechanism to induce the THz radiation in our symmetry-broken $PtTe_2$ films.

Supplementary Note 4: Symmetry analysis for the helicity dependent THz emission in defect-gradient $PtTe_2$ films

The electromagnetic radiation in the THz spectral range (1 THz = 1 ps^{-1}) is generated by changes of the current density ($\mathbf{J}$ ($\mathbf{r}$, t)) on a picosecond timescale according to the Maxwell equations:[14,15]

$$\mathbf{E}_{\mathrm{rad}}(\mathbf{r},t)=-\frac{\partial\mathbf{A}}{\partial t}=-\frac{1}{4\pi\epsilon_0 c^2}\int\frac{1}{|\mathbf{r}-\mathbf{r}'|}\frac{\partial\mathbf{J}(\mathbf{r}',t')}{\partial t}d\mathbf{r}' \quad (2)$$

where $\mathbf{A}$ is the electromagnetic vector potential, ϵ_0 is the free-space permittivity, and

$t' = t - \frac{|\mathbf{r}-\mathbf{r}'|}{c}$ is the retarded time between the source location ($\mathbf{r}'$) and measurement location ($\mathbf{r}$) for a radiation field propagating at the light speed in vacuum (c).

Here, we provide the more detailed in-plane symmetry analysis of the photocurrent in the $PtTe_2$ films. Phenomenologically, the CPGE photocurrent (J) and the CPGE tensor ($\beta(\omega)$) can be respectively described as[16-18]

$$J_\lambda^{\mathrm{CPGE}} = i\sum_\mu \beta_{\lambda\mu}\left(\mathbf{E}\times\mathbf{E}^*\right)_\mu \text{ and } \beta_{\lambda\mu}(\omega) = \sum_{\mathbf{k}} \tilde{\beta}_{\lambda\mu}(\mathbf{k},\omega) \tag{3}$$

where $\mathbf{E}^*(\omega) = \mathbf{E}(-\omega)$, which is the electric field of the circularly polarized light. λ and μ indices are the photocurrent direction and the circularly polarized light direction, respectively. And $\tilde{\beta}_{\lambda\mu}(\mathbf{k},\omega) = \epsilon_{\mu\nu\xi}\Gamma_{\lambda\nu\xi}(\mathbf{k},\omega)$. Here,

$$\Gamma_{\lambda\nu\xi}(\mathbf{k},\omega) = \frac{\pi e^3}{\hbar^2 V}\sum_{n,m} f_{nm}^{\mathbf{k}}\Delta_{\mathbf{k},nm}^{\lambda} r_{\mathbf{k},nm}^{\upsilon} r_{\mathbf{k},mn}^{\xi}\delta\left(\hbar\omega - E_{\mathbf{k},mn}\right) \tag{4}$$

where V is the sample volume. $E_{\mathbf{k},mn} = E_{\mathbf{k},m} - E_{\mathbf{k},n}$ and $f_{nm}^{\mathbf{k}} = f_n^{\mathbf{k}} - f_m^{\mathbf{k}}$ are differences between band energies and Fermi-Dirac distributions, respectively. The other terms in Supplementary Equation (4) are expressed *via* $\Delta_{\mathbf{k},nm}^{\lambda} = \partial_{k_\lambda}E_{\mathbf{k},mn}/\hbar$ and $r_{\mathbf{k},nm}^{\lambda} = i\langle m(\mathbf{k})|\partial_{k_\lambda}|n(\mathbf{k})\rangle$. The latter equation is the cross gap Berry connection. For a nonmagnetic material with a crystal symmetry g, the relationship of $\Delta_{g\mathbf{k},nm}^{\lambda}$ and $r_{g\mathbf{k},nm}^{\lambda}$ between $\mathbf{k}$ and $g\mathbf{k}$ is written as

$$\Delta_{g\mathbf{k},nm}^{\lambda} = \sum_{\lambda'}\frac{\partial(g\mathbf{k})_{\lambda'}}{\partial k_\lambda}\Delta_{\mathbf{k},nm}^{\lambda'} \text{ and } r_{g\mathbf{k},nm}^{\lambda} = \sum_{\lambda'}\frac{\partial(g\mathbf{k})_{\lambda'}}{\partial k_\lambda} r_{\mathbf{k},nm}^{\lambda'} \tag{5}$$

By substituting Supplementary Equation (4) and (5) into $\tilde{\beta}_{\lambda\mu}(\mathbf{k},\omega) = \epsilon_{\mu\nu\xi}\Gamma_{\lambda\nu\xi}(\mathbf{k},\omega)$, we can obtain the relationship of $\tilde{\beta}_{\lambda\mu}(\mathbf{k},\omega)$ and $\tilde{\beta}_{\lambda\mu}(g\mathbf{k},\omega)$ connected by g:

$$\tilde{\beta}_{\lambda\mu}(g\mathbf{k},\omega) = \epsilon_{\mu\nu\xi}\sum_{\lambda',\upsilon',\xi'}\frac{\partial(g\mathbf{k})_{\lambda'}}{\partial k_\lambda}\frac{\partial(g\mathbf{k})_{\upsilon'}}{\partial k_\upsilon}\frac{\partial(g\mathbf{k})_{\xi'}}{\partial k_\xi}\Gamma_{\lambda'\upsilon'\xi'}(\mathbf{k},\omega) \tag{6}$$

The above description is a general case without mentioning the specific crystal symmetry. In the following, we shall discuss the relationship between the CPGE photocurrent and crystal symmetry in the different symmetry point group in the defect-gradient $PtTe_2$ films.

(i) D_{3d} symmetry

Bulk defect-free $PtTe_2$ crystal is centrosymmetric as the inversion center is located at Pt sites. From the perspective of Pt atoms, $PtTe_2$ has the structure of D_{3d} point group, where the symmetry operations include identity (I), threefold rotation axis (C_3), horizontal mirror plane (σ_h), and three perpendicular two-fold rotation axes (C_2). Due to the characteristics of AA stacking between neighboring layers, the symmetry elements are not reduced in 2D layered crystals that we concern. If the $PtTe_2$ has the point group of the D_{3d} (centrosymmetric), the tensor $\tilde{\beta}_{\lambda\mu}(D\mathbf{k},\omega)$ becomes

$$\begin{aligned}\tilde{\beta}_{\lambda\mu}(D\mathbf{k},\omega) &= \epsilon_{\mu\nu\xi}\sum_{\lambda',\nu',\xi',}\frac{\partial(D\mathbf{k})_{\lambda'}}{\partial k_\lambda}\frac{\partial(D\mathbf{k})_{\nu'}}{\partial k_\nu}\frac{\partial(D\mathbf{k})_{\xi'}}{\partial k_\xi}\Gamma_{\lambda'\nu'\xi'}(\mathbf{k},\omega)\\ &= \epsilon_{\mu\nu\xi}\frac{\partial(D\mathbf{k})_{\lambda}}{\partial k_\lambda}\frac{\partial(D\mathbf{k})_{\nu}}{\partial k_\nu}\frac{\partial(D\mathbf{k})_{\xi}}{\partial k_\xi}\Gamma_{\lambda\nu\xi}(\mathbf{k},\omega)\\ &= -\epsilon_{\mu\nu\xi}\Gamma_{\lambda\nu\xi}(\mathbf{k},\omega) = -\tilde{\beta}_{\lambda\mu}(\mathbf{k},\omega) \end{aligned} \quad (7)$$

where the second line uses $D\mathbf{k} = (-k_x, -k_y, -k_z)$. Due to CPGE tensor $\beta_{\lambda\mu}(\omega) = \sum_{\mathbf{k}}\tilde{\beta}_{\lambda\mu}(\mathbf{k},\omega)$, we obtain $\beta_{\lambda\mu}(\omega) = 0$. Therefore, the CPGE tensor $\beta_{\lambda\mu}(\omega)$ becomes zero in inversion symmetric systems. The symmetry breaking in the system is a prerequisite for generating a non-zero in-plane CPGE tensor which is responsible for the CPGE photocurrent.[18,19] Since we observe a nonzero second-order nonlinear conductivity and a finite photocurrent, the symmetry point group of $PtTe_2$ should reduce from D_{3d} (centrosymmetric) to C_{3v} (non-centrosymmetric). Therefore, the D_{3d} symmetry must be broken in our films.

(ii) C_{3v} symmetry

From the perspective of Te atoms, layered $PtTe_2$ has the structure of C_{3v} point group, where symmetry operations include identity (I), threefold rotation axis (C_3), and three vertical mirror planes (σ_v). In the case of the V_{Te} defect, one Te atom is removed from a $PtTe_2$ layer. Subsequently, three Pt atoms surrounding the vacancy are relaxed, resulting in the closer distance among them. Thus, the atom position surrounding the vacancy slightly changes from their pristine positions.[20] Considering the structural relaxation, it may still maintain the C_{3v} symmetry of the V_{Te}. A three-fold rotation symmetry maintains around the z axis, and three mirror planes are perpendicular to the xy plane (Supplementary Fig. 21a). The CPGE photocurrent for the C_{3v} symmetry can be given by:

$$\mathbf{J}^{\mathrm{CPGE}} = i\beta(\mathbf{E}\times\mathbf{E}^*) \quad (8)$$

Under a 2π/3 rotation

$$R = \begin{pmatrix} \cos(2\pi/3) & \sin(2\pi/3) & 0\\ -\sin(2\pi/3) & \cos(2\pi/3) & 0\\ 0 & 0 & 1\end{pmatrix} \quad (9)$$

Thus, $(\mathbf{E}\times\mathbf{E}^*)$ and $\mathbf{J}^{\mathrm{CPGE}}$ become $R\,(\mathbf{E}\times\mathbf{E}^*)$ and $R\mathbf{J}^{\mathrm{CPGE}}$, respectively. Since β should remain the same under the rotation symmetry, we obtain

$$R\mathbf{J}^{CPGE} = i\beta R(\mathbf{E}\times\mathbf{E}^*) \tag{10}$$

By replacing Supplementary Equation (10) into Supplementary Equation (8), we obtain

$$R\beta = \beta R \tag{11}$$

The mirror symmetry can provide an additional constraint. The angle between the mirror plane and the x axis is defined as ψ, which can be characterized by the operator

$$M = \begin{pmatrix} \cos(2\psi) & \sin(2\psi) & 0 \\ \sin(2\psi) & -\cos(2\psi) & 0 \\ 0 & 0 & 1 \end{pmatrix} \tag{12}$$

When $\mathbf{J}^{\mathrm{CPGE}}$ becomes $M\mathbf{J}^{\mathrm{CPGE}}$, β should remain constant. As a pseudo-vector, $(\mathbf{E}\times\mathbf{E}^*)$ becomes $-M\,(\mathbf{E}\times\mathbf{E}^*)$. Hence, we obtain

$$M\beta = -\beta M \tag{13}$$

Considering the combined constraints of rotational and mirror symmetries, $\beta_{\lambda\mu}$ should have only one independent parameter:

$$\beta_{\lambda\mu} = \begin{pmatrix} 0 & \beta_{xy} & 0 \\ -\beta_{xy} & 0 & 0 \\ 0 & 0 & 0 \end{pmatrix} \tag{14}$$

and the CPGE photocurrent should have a form of

$$\mathbf{J}^{\mathrm{CPGE}} = i\begin{pmatrix} 0 & \beta_{xy} & 0 \\ -\beta_{xy} & 0 & 0 \\ 0 & 0 & 0 \end{pmatrix}\begin{pmatrix} (\mathbf{E}\times\mathbf{E}^*)_x \\ (\mathbf{E}\times\mathbf{E}^*)_y \\ (\mathbf{E}\times\mathbf{E}^*)_z \end{pmatrix} = i\begin{pmatrix} \beta_{xy}((\mathbf{E}\times\mathbf{E}^*)_y) \\ -\beta_{xy}((\mathbf{E}\times\mathbf{E}^*)_x) \\ 0 \end{pmatrix} \tag{15}$$

For a light sheds in the y-z plane, $(\mathbf{E}\times\mathbf{E}^*)_x = 0$. Therefore, the photocurrent only flows along its x direction. Moreover, for an incident angle of α, $i(\mathbf{E}\times\mathbf{E}^*)_y = |E|^2 P sin\alpha$, where P represents the helicity of the light and $P = \sin(2\theta)$. Here, θ is the angle of the quarter-wave plate. Therefore, we obtain

$$J^{\mathrm{CPGE}} \propto \sin(2\theta)\sin(\alpha) \tag{16}$$

This relation turns out the nonzero CPGE photocurrent, which has a $\sin(2\theta)$ dependence in the polarization experiment. Furthermore, only nonzero CPGE photocurrent is produced for a nonzero incident angle α. However, in our measurements, the nonzero CPGE photocurrent is obtained under normal incidence (Fig. 2c). Therefore, the C_{3v} symmetry should be reduced to C_{1v}.

(iii) C_{1v} symmetry

Let us consider an even lower symmetry case, namely a V_{Te}-gradient $PtTe_2$ system that has only one mirror plane perpendicular to the xy plane. Likewise, we introduce the angle ψ here, and under the mirror reflection, $\mathbf{J}^{\mathrm{CPGE}}$ becomes $M\mathbf{J}^{\mathrm{CPGE}}$, where M has been defined in Supplementary Equation (12). As a pseudo-vector, $(\mathbf{E}\times\mathbf{E}^*)$

becomes $-M(\mathbf{E}\times\mathbf{E}^*)$ again, and we also obtain the Supplementary Equation (13) of $M\beta = -\beta M$. Such an absence of the 2π/3 rotational symmetry makes more independent parameters emerge in $\beta_{\lambda\mu}$. The CPGE photocurrent for the C_{1v} symmetry thus takes the form

$$\mathbf{J}^{\mathrm{CPGE}} = i\begin{pmatrix} 0 & \beta_{xy} & \beta_{xz} \\ -\beta_{xy} & 0 & \beta_{yz} \\ \beta_{zx} & \beta_{zy} & 0 \end{pmatrix}\begin{pmatrix} (\mathbf{E}\times\mathbf{E}^*)_x \\ (\mathbf{E}\times\mathbf{E}^*)_y \\ (\mathbf{E}\times\mathbf{E}^*)_z \end{pmatrix}$$
$$\propto \begin{pmatrix} [\beta_{xy}\sin(\varphi)\sin(\alpha) + \beta_{xz}\cos(\alpha)]\sin(2\theta) \\ [-\beta_{xy}\cos(\varphi)\sin(\alpha) + \beta_{yz}\cos(\alpha)]\sin(2\theta) \\ 0 \end{pmatrix} \quad (17)$$

where we assume that the J_z^{CPGE} component should be zero for a defect-gradient film, and φ is the sample azimuthal angle. The mirror symmetry turns out the additional conditions:

$$\beta_{xz} = \frac{1-\cos(2\psi)}{\sin(2\psi)}\beta_{yz} \quad (18)$$

$$\beta_{zx} = \frac{1-\cos(2\psi)}{\sin(2\psi)}\beta_{zy} \quad (19)$$

Thus, we obtain a CPGE contribution independent on θ and it changes with $\cos\alpha$. In this case, the V_{Te} defect is required to be generated symmetrically on both sides of a mirror symmetry axis (see Supplementary Fig. 21b), which is very difficult to control during the preparation of large-area thin films. Thus, the symmetry of the system is further lowered to C_1.

(iv) C_1 symmetry

The $PtTe_2$ films containing the V_{Te} defect can also maintain the C_{3v} symmetry due to the relaxation of the atoms. However, the discrepancy in adjacent monolayers naturally breaks the in-plane inversion symmetry reduced from C_{3v} to C_1 (Supplementary Fig. 21c). For the C_1 symmetry, there is no symmetry operation. Therefore, the nonzero elements of $\beta_{\lambda\mu}$ take the form:

$$\beta_{\lambda\mu} = \begin{pmatrix} \beta_{xx} & \beta_{xy} & \beta_{xz} \\ \beta_{yx} & \beta_{yy} & \beta_{yz} \\ \beta_{zx} & \beta_{zy} & \beta_{zz} \end{pmatrix} \quad (20)$$

Thus, the CPGE photocurrent for the C_1 symmetry takes the form

$$\mathbf{J}^{\mathrm{CPGE}} = i\begin{pmatrix} \beta_{xx} & \beta_{xy} & \beta_{xz} \\ \beta_{yx} & \beta_{yy} & \beta_{yz} \\ \beta_{zx} & \beta_{zy} & \beta_{zz} \end{pmatrix}\begin{pmatrix} (\mathbf{E}\times\mathbf{E}^*)_x \\ (\mathbf{E}\times\mathbf{E}^*)_y \\ (\mathbf{E}\times\mathbf{E}^*)_z \end{pmatrix}$$
$$\propto \begin{pmatrix} [\beta_{xx}\cos\varphi\sin\alpha + \beta_{xy}\sin\varphi\sin\alpha + \beta_{xz}\cos\alpha]\sin(2\theta) \\ [\beta_{yx}\cos\varphi\sin\alpha + \beta_{yy}\sin\varphi\sin\alpha + \beta_{yz}\cos\alpha]\sin(2\theta) \\ 0 \end{pmatrix} \quad (21)$$

Apparently, the CPGE photocurrent is still nonzero under normal incidence with the symmetry reduced to C_1. The observation of only C_1 symmetry is seen from the sample-azimuthal-angle (φ)-dependent THz radiation under the linearly polarized

excitation (Supplementary Fig. 21d,e).

In addition, the recently reported out-of-plane composition gradient has been evidenced that it can also simultaneously break both out-of-plane and in-plane symmetries, enabling in-plane-current to scale the out-of-plane magnetization switching.[21-26] The construction of symmetry-mismatched heterointerfaces, such as WSe_2/SiP, can also break the in-plane symmetry (reduced to C_{1v}), thus generating the in-plane CPGE photocurrents.[27] These reports are consistent with our observation of in-plane THz electric field scaling of the out-of-plane defect gradient in $PtTe_2$ films.

Overall, with the above derivation and the associated experimental evidence, it is established that the vertical defect gradient not only breaks the out-of-plane inversion symmetry, but also breaks the in-plane C_{3v} symmetry (reduced to C_1). Note that recent studies have also shown that inversion symmetry breaking as well as C_3 rotational symmetry breaking can generate the asymmetric distribution of Berry curvature, thus enabling the non-vanishing Berry curvature dipole (BCD) and various quantum geometrical phenomena.[28,29]

Supplementary Note 5: Two-band model fitting for the nonlinear characteristic of the Hall resistivity in defect-gradient $PtTe_2$ films

Recent study has revealed that the V_{Te} concentration has a significant influence on the band structure in $PtTe_2$ films.[30] The nonlinear Hall characteristic is observed in our as-grown $PtTe_2$ films as compared with that of the Te-passivated sample (Supplementary Fig. 18d), which indicates that the multiple types of carriers participate in the conductivity due to the modulation of the energy band near the Fermi level by V_{Te}. Therefore, we can obtain the carrier density and mobility by a two-band model fitting. In order to quantitatively analyze the experimental data, the measured ρ_{xx} and ρ_{xy} at low temperatures can be converted to the conductivity:

$$\sigma_{xx} = \rho_{xx}/(\rho_{xx}^2 + \rho_{xy}^2) \tag{22}$$

$$\sigma_{xy} = \rho_{xy}/(\rho_{xx}^2 + \rho_{xy}^2) \tag{23}$$

Next, $\sigma_{xx}\ (B)$ and $\sigma_{xy}\ (B)$ are both fitted by the formula:

$$\sigma_{xx}(B) = e(\frac{n_h\mu_h}{1+(\mu_h B)^2} + \frac{n_e\mu_e}{1+(\mu_e B)^2}) \tag{24}$$

$$\sigma_{xy}(B) = eB(\frac{n_h\mu_h^2}{1+(\mu_h B)^2} - \frac{n_e\mu_e^2}{1+(\mu_e B)^2}) \tag{25}$$

And we can get the effective carrier contribution for the Hall nonlinear characteristic:

$$\mathrm{V_{Te}\ contribution} = \frac{n_h\mu_h}{n_e\mu_e+n_h\mu_h} \times 100\% \tag{26}$$

where n_e and μ_e , n_h and μ_h represent the carrier concentrations and the carrier mobilities of electron- and hole-like bands, respectively. The introduction of the V_{Te} defect leads to the nonlinear characteristic of Hall curves, which contributes to the hole carrier conduction. Thus, we use the V_{Te} contribution expression (Supplementary Equation (26)) as the quantification of the effective vacancy defect participating in the transport. The detailed fitting processes are shown in Supplementary Fig. 18e,f. The

fitting results from Supplementary Fig. 18b and Fig. 4b are displayed in Supplementary Tables 2 and 4, respectively.

Supplementary Note 6: The connection between Berry curvature and the CPGE

Berry curvature has an important impact on the CPGE because of the optical selection rule over circular polarizations. Here, we provide the derivation between the BCD and the CPGE photocurrent according to the previous reports.[31,32]

The CPGE generation under normal incidence consists of two steps: (i) circularly polarized light excites interband transitions that obeys an optical selection rule; (ii) the optically excited electrons and holes propagate at different group velocities, thus producing a nonzero CPGE photocurrent, which is mathematically described by

$$\mathbf{J}^{\mathrm{CPGE}} = -\frac{2\pi e\tau}{\hbar}\sum_{I,F}\int\frac{d^2\mathbf{k}}{(2\pi)^2}[\Delta\vec{v}(\mathbf{k})]\gamma(\mathbf{k})\delta[\Delta\varepsilon(\mathbf{k}) - \hbar\omega][\Delta f(\mu,\mathbf{k})] \tag{27}$$

where $\mathbf{J}^{\mathrm{CPGE}}$ is the difference between the RCP and LCP photocurrents, τ is the relaxation time, $\gamma(\mathbf{k}) = |P^{\mathrm{RCP}}(\mathbf{k})|^2 - |P^{\mathrm{LCP}}(\mathbf{k})|^2$ is the difference between the optical transition probabilities for RCP and LCP light, I and F are all initial state and final state that comply with the energy conservation restricted by the δ function, and $\Delta\vec{v}$, $\Delta\varepsilon(\mathbf{k})$, and $\Delta f(\mu,\mathbf{k})$ are the difference of the group velocity, energy, and Fermi-Dirac distribution between the initial state and final state, respectively. P is the dipole of optical transition, which is expressed by

$$P = \frac{e}{m_e}\langle F|\mathbf{A}\cdot\mathbf{p}|I\rangle \tag{28}$$

where e is the charge, m_e is the electron mass, $|I>$ and $|F>$ are the wavefunctions of all possible initial state and final state, respectively, **A** is the light vector potential, and **p** is the momentum operator, which takes the form

$$\mathbf{p} = \frac{m_e}{i\hbar}[\mathbf{r}, H] \tag{29}$$

Using Supplementary Equation (28), $\gamma(\mathbf{k})$ under normal incidence and circularly polarized light is described by

$$\begin{aligned}\gamma(\mathbf{k}) &= |P^{\mathrm{RCP}}(\mathbf{k})|^2 - |P^{\mathrm{LCP}}(\mathbf{k})|^2 \\ &= (\tfrac{Ae}{m_e})^2[|\langle F|(p_x + ip_y)|I\rangle|^2 - |\langle F|(p_x - ip_y)|I\rangle|^2] \\ &= \frac{2A^2e^2}{m_e^2}[i\langle F|p_y|I\rangle\langle I|p_x|F\rangle - i\langle F|p_x|I\rangle\langle I|p_y|F\rangle]\end{aligned} \tag{30}$$

where $|I>$ and $|F>$ are the Bloch wavefunctions of all possible initial state and final state for the interband transition, p_x and p_y are the momentum operators in the x,y direction, and A is the magnitude of **A**.

In 2D systems, the Berry curvature is only along the vertical direction. For an N-band system it is defined as

$$\Omega_z^{\text{band } n}(\mathbf{k}) = i\sum_{n'\neq n}\frac{\left\langle n'\left|\frac{\partial H}{\partial k_x}\right|n\right\rangle\left\langle n\left|\frac{\partial H}{\partial k_y}\right|n'\right\rangle - \left\langle n'\left|\frac{\partial H}{\partial k_y}\right|n\right\rangle\left\langle n\left|\frac{\partial H}{\partial k_x}\right|n'\right\rangle}{(\varepsilon_{n'}-\varepsilon_n)^2} \tag{31}$$

It is seen that the interband transition in the complicated CPGE process involves a number of bands when the photon energy $\hbar\omega$ is large, Accordingly, one needs to sum over all possible initial state and final state that obey the energy conservation ($\sum_{I,F}$ in Supplementary Equation (27)).

In a two-band system, many previous studies[24,31,33-35] demonstrated that $\gamma(\vec{k})$ can be written *via* the Berry curvature:

$$\gamma(\mathbf{k}) = \frac{2e^2}{\hbar^2}A^2\Omega_z^{\text{NBC}}(\mathbf{k})[\Delta\varepsilon(\mathbf{k})]^2 \tag{32}$$

Here, the negative and positive Berry curvatures are denoted as NBC and PBC, respectively. The momentum operator p_x and p_y in the P (optical transition dipole) is calculated by $\frac{m_e}{\hbar}\frac{\partial H}{\partial k_i}$ through the Peierls substitution.[24,33-36] Hence, in a two-band system, the Berry curvature in Supplementary Equation (31) is simplified as

$$\Omega_z^{\text{PBC}}(\mathbf{k}) = -\Omega_z^{\text{NBC}}(\mathbf{k}) = i\frac{\left\langle v\left|\frac{\partial H}{\partial k_x}\right|c\right\rangle\left\langle c\left|\frac{\partial H}{\partial k_y}\right|v\right\rangle - \left\langle v\left|\frac{\partial H}{\partial k_y}\right|c\right\rangle\left\langle c\left|\frac{\partial H}{\partial k_x}\right|v\right\rangle}{(\Delta\varepsilon)^2} \tag{33}$$

where $|v>$ and $|c>$ are the wavefunctions of the only PBC band and the only NBC band, respectively. It is seen that the Berry curvature of the PBC ($\Omega_z^{\text{PBC}}(\mathbf{k})$) is solely contributed by the NBC band, and vice versa (Supplementary Equation (33)). While in an N-band system, the Berry curvature of the nth band ($\Omega_z^n(\mathbf{k})$) is contributed by all the other $N-1$ bands (Supplementary Equation (31)). With the two-band approximation and the substitution of $p_i \rightarrow \frac{m_e}{\hbar}\frac{\partial H}{\partial k_i}$, $\gamma(\mathbf{k})$ can be described by

$$\gamma(\mathbf{k}) = \frac{2e^2}{\hbar^2}A^2\Omega_{\text{z}}^{\text{NBC}}(\mathbf{k})[\Delta\varepsilon(\mathbf{k})]^2 \tag{34}$$

We now see that optical transition probability is directly proportional to the Berry curvature. The PBC and NBC states can selectively absorb the RCP and LCP light, respectively. The similar case has been reported previously.[24,31,33-36]

Next, we show the derivation of the connection between the CPGE photocurrent (Supplementary Equation (27)) and the BCD.

$$J_x^{\text{CPGE}} = -\frac{2\pi e\tau}{\hbar}\sum_{I,F}\int\frac{d^2\mathbf{k}}{4\pi^2}[\Delta\mathbf{v}(\mathbf{k})]\gamma(\mathbf{k})\delta[\Delta\varepsilon(\mathbf{k})-\hbar\omega][\Delta f(\mu,\mathbf{k})]$$

$$= -\frac{e^3\tau A^2}{\pi\hbar^3}\int dk_x dk_y\frac{d(\Delta\varepsilon)}{\hbar dk_x}\Omega(\mathbf{k})(\Delta\varepsilon)^2\delta(\Delta\varepsilon-\hbar\omega)$$

$$= -\frac{e^3\tau A^2}{\pi\hbar^4}\int dk_y d(\Delta\varepsilon)\,\Omega(\mathbf{k})(\Delta\varepsilon)^2\delta(\Delta\varepsilon-\hbar\omega)$$

$$= -\frac{e^3 \tau E^2}{\pi \hbar^2} \oint dk_y \, \Omega(\mathbf{k}) \tag{35}$$

where $\oint dk_y$ is the closed loop integral defined along the k-contours corresponding to an $\hbar\omega$ interband transition, and E and A are the magnitudes of the electric field and the light vector potential, respectively. In the above derivation, the definition of group velocity $\Delta\mathbf{v}(\mathbf{k}) = \frac{1}{\hbar} \nabla_{\mathbf{k}}(\Delta\varepsilon)$ is applied, and we assume $\Delta f(\mu, \mathbf{k}) = 1$. The summation ($\sum_{I,F}$) is dropped because there are only two initial state and final state in the two-band system. Given that the Berry curvature in 2D systems only holds the vertical component, the CPGE photocurrent can be rewritten as:

$$\mathbf{J}^{\mathrm{CPGE}} = \frac{e^3 \tau}{\pi \hbar^2} \mathrm{Im}[\mathbf{E}(-\omega) \times \hat{c} \, (\mathbf{\Lambda}^{\Omega} \cdot \mathbf{E}(\omega))] \tag{36}$$

where $\mathbf{\Lambda}^{\Omega} = \oint d\mathbf{k} \times \mathbf{\Omega}(\mathbf{k})$ is rightly the BCD, $\mathbf{E}(\omega) = (E_x e^{i\omega t}, E_y e^{i(\omega t + \varsigma)}, 0)$ denotes the electric field of the normal incident light with a generic polarization, E_x and E_y are the electric fields in the x,y direction, and ς is the phase difference between the x and y components. When $\varsigma = \pm\frac{\pi}{2}$, $E_x = E_y$ corresponds to the normal incident RCP and LCP light.

During our calculations for the $PtTe_2$-based system, Wannier90 was used to fit the DFT band structures. The home-made codes were developed to obtain the corresponding effective Hamiltonian. We then calculated the subband-dependent Berry curvature and the energy-dependent BCD (Λ_x^{Ω}). Based on this process, we obtain the calculation results for the microscopic origin of helicity dependent THz emission (Fig. 4d-f).

Supplementary Figures

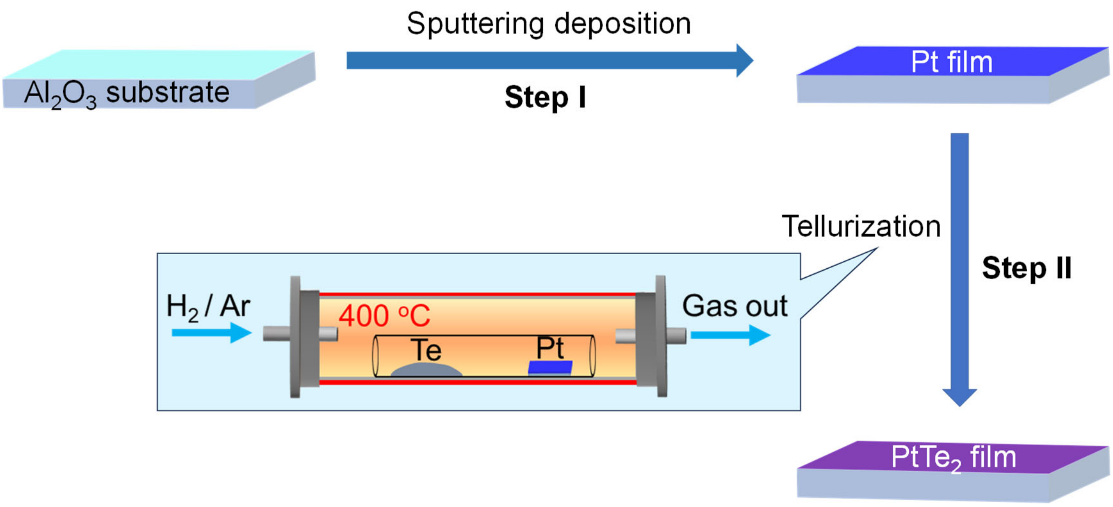

Supplementary Figure 1. Schematic illustration of the modified two-step CVD growth process.

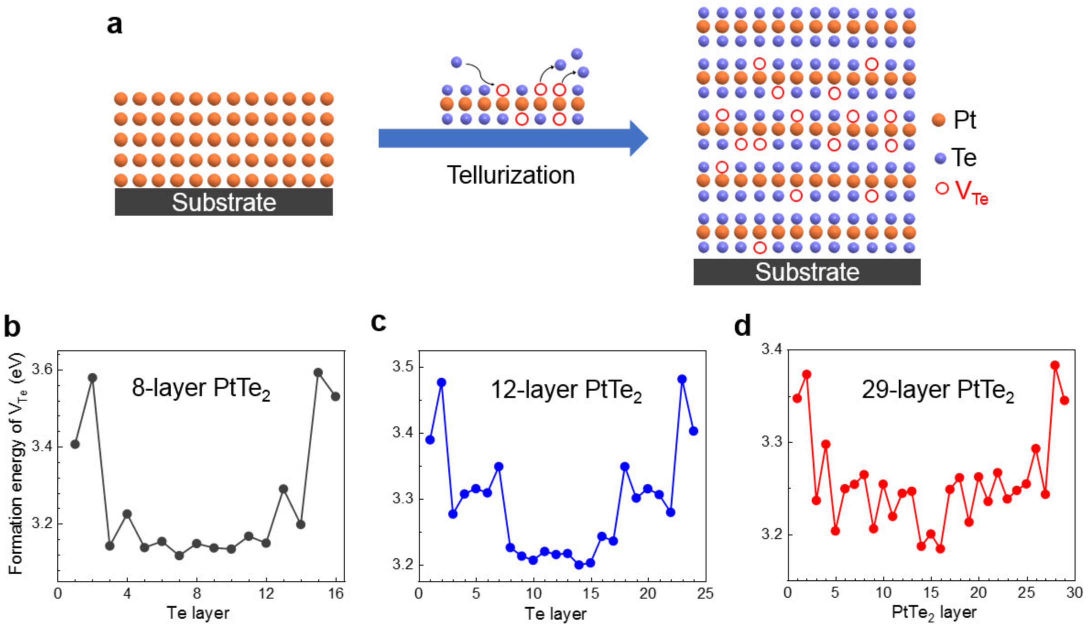

Supplementary Figure 2. The depth-profile formation energy of the V_{Te} defect from the substrate to the vacuum. a, Schematic illustration the V_{Te} formation process. **b-d**, The calculated formation energy of V_{Te} in $PtTe_2$ films with the varied thickness from DFT calculations based on the growth dynamics. We adopt each Te atomic layer in **b,c** and each $PtTe_2$ layer in **d** in DFT calculations.

Note: The formation energy under the condition of the V_{Te} defect here is calculated by $E_f = E_{tot}(\text{with defect}) + N\mu - E_{tot}(\text{without defect})$, where E_{tot} (with defect) and E_{tot} (without defect) are the total energies of the defective and defect-free supercell, respectively; μ and N represent the chemical potential and the number of V_{Te}, respectively. The μ is estimated by $\mu = (E_{PtTe2} - M \times \mu_{Pt})/K$, where M and K denote the numbers of Pt and Te atoms, respectively.

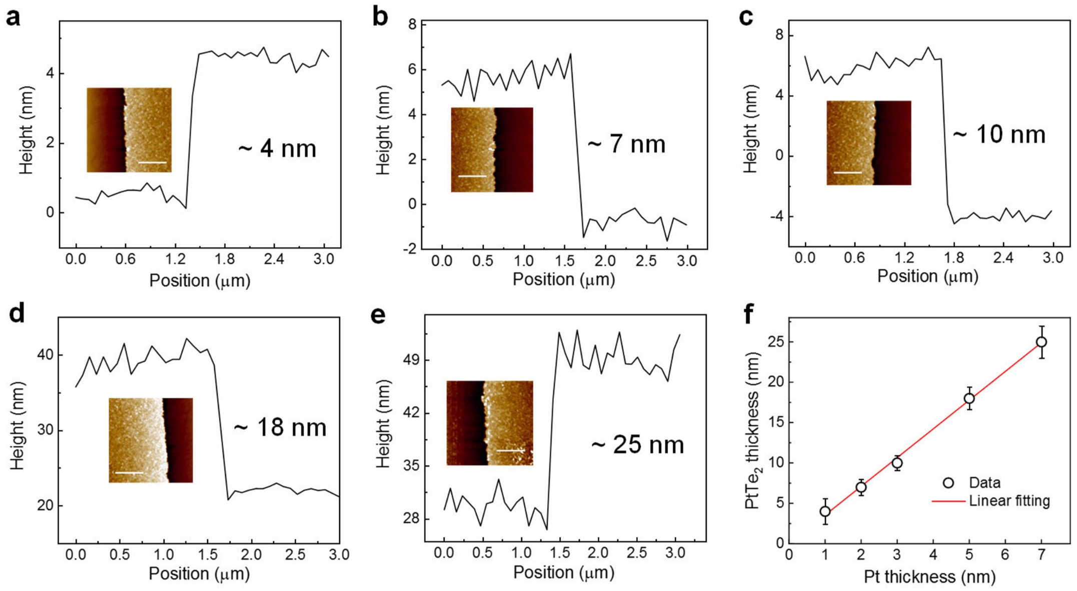

Supplementary Figure 3. Thickness determination of various $PtTe_2$ films by atomic force microscopy (AFM). a-e, The height profiles of various $PtTe_2$ films grown on Al_2O_3 substrates, which is determined to be about 4, 7, 10, 18 and 25 nm from AFM images (insets), respectively. The scale bars of the AFM images are all 1 μm. **f**, The linear relationship between the $PtTe_2$ thickness and the Pt thickness, indicating the reproducibility of the two-step CVD growth process. The error bars indicate the uncertainty from AFM measurements.

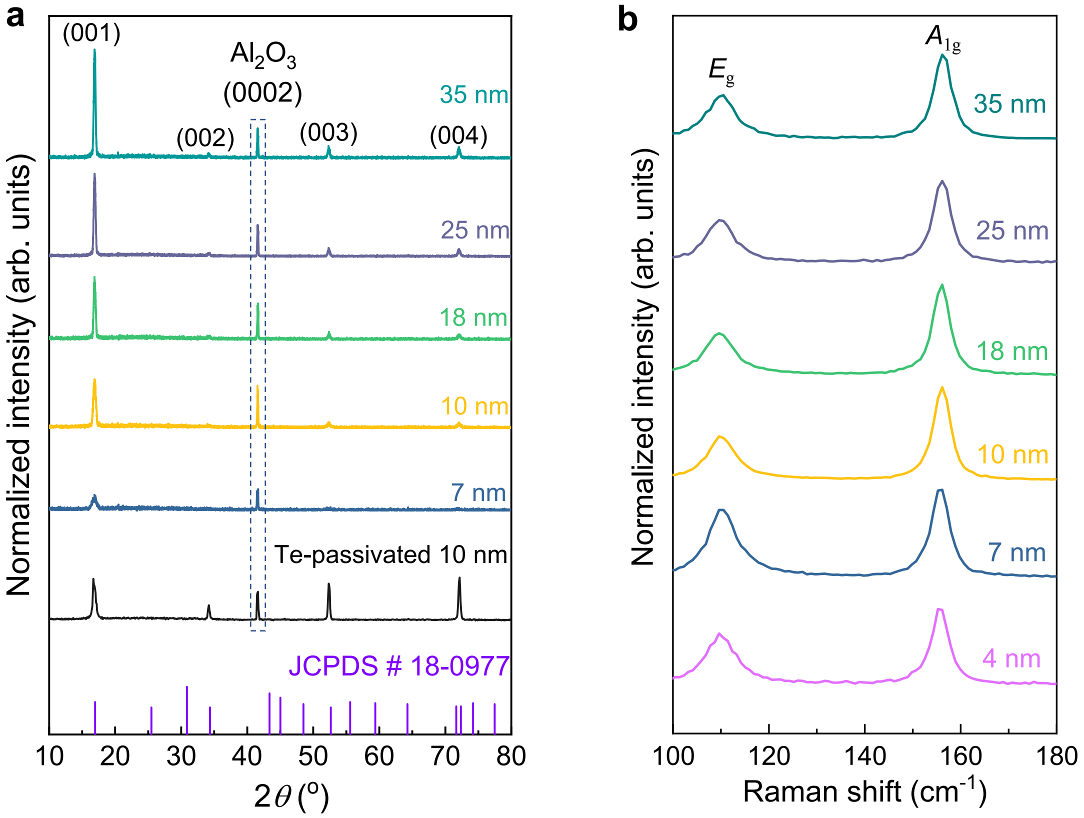

Supplementary Figure 4. XRD patterns and Raman spectra of $PtTe_2$ films with various thicknesses. a, XRD patterns. The 10-nm-thick Te-passivated sample is also included. The XRD patterns exhibit the pronounced (00*l*) characteristic diffraction peaks for all films. Such consistent orientations of the diffraction peaks indicate the excellent crystallinity. Meanwhile, it can be found that the crystallinity is improved by the post-annealing process in the Te vapor, and the positions of the diffraction peaks remain unchanged. **b**, Room-temperature micro-Raman spectra with typically E_g and A_{1g} vibrational modes at about 111.4 and 157.6 cm^{-1}, respectively.

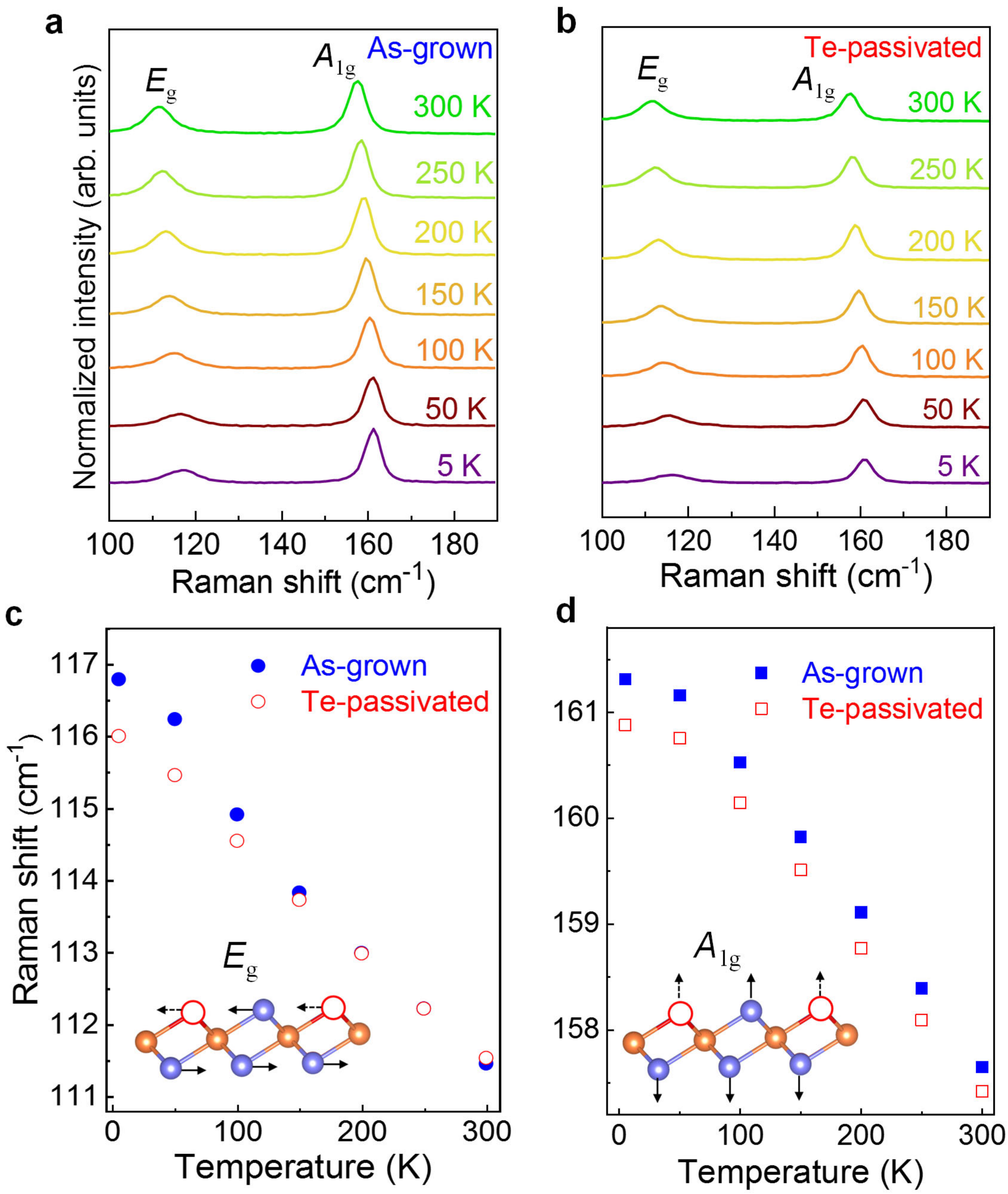

Supplementary Figure 5. Temperature-dependent Raman spectra of the as-grown and Te-passivated $PtTe_2$ films with the thickness of 10 nm. a,b, Temperature-dependent Raman spectra for the as-grown and Te-passivated samples, respectively. **c,d**, Temperature evolution of the extracted E_g and A_{1g} modes of the as-grown and Te-passivated samples from **a** and **b**, respectively. The insets show the schematic diagrams of Te atoms' vibration for E_g and A_{1g} modes, respectively, in which the V_{Te} defect is indicated. The discussion can be found in Supplementary Note 2.

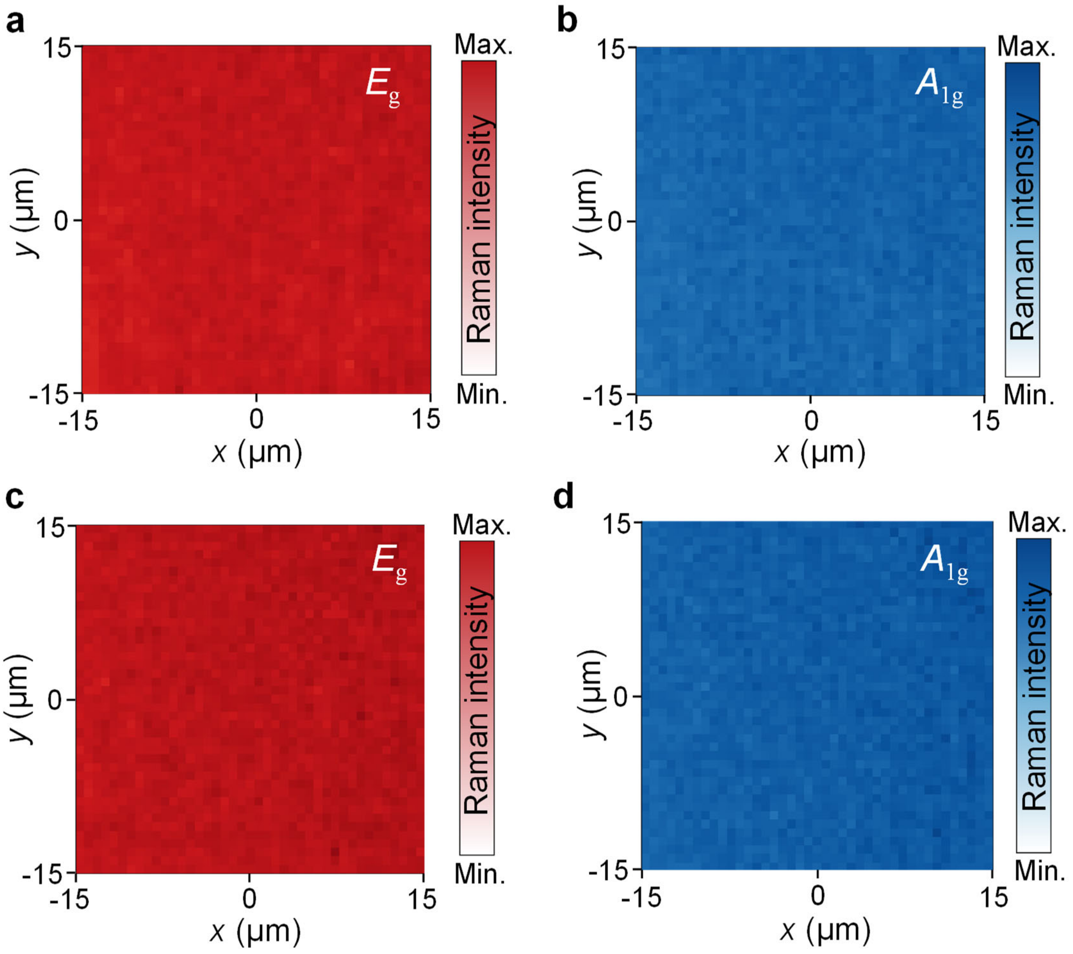

Supplementary Figure 6. Raman mapping images from two different regions (with an interval of about 1 mm) on the single $PtTe_2$ film with the thickness of 10 nm. The Raman mapping images with a micrometer resolution are plotted with the intensity of in-plane E_g (**a**,**c**) and out-of-plane A_{1g} vibrational modes (**b**,**d**), respectively, indicating that the as-grown $PtTe_2$ films containing the vertical V_{Te} defect gradient (as determined by the quantitative electron ptychography) are largely uniform along the surface on a millimeter scale.

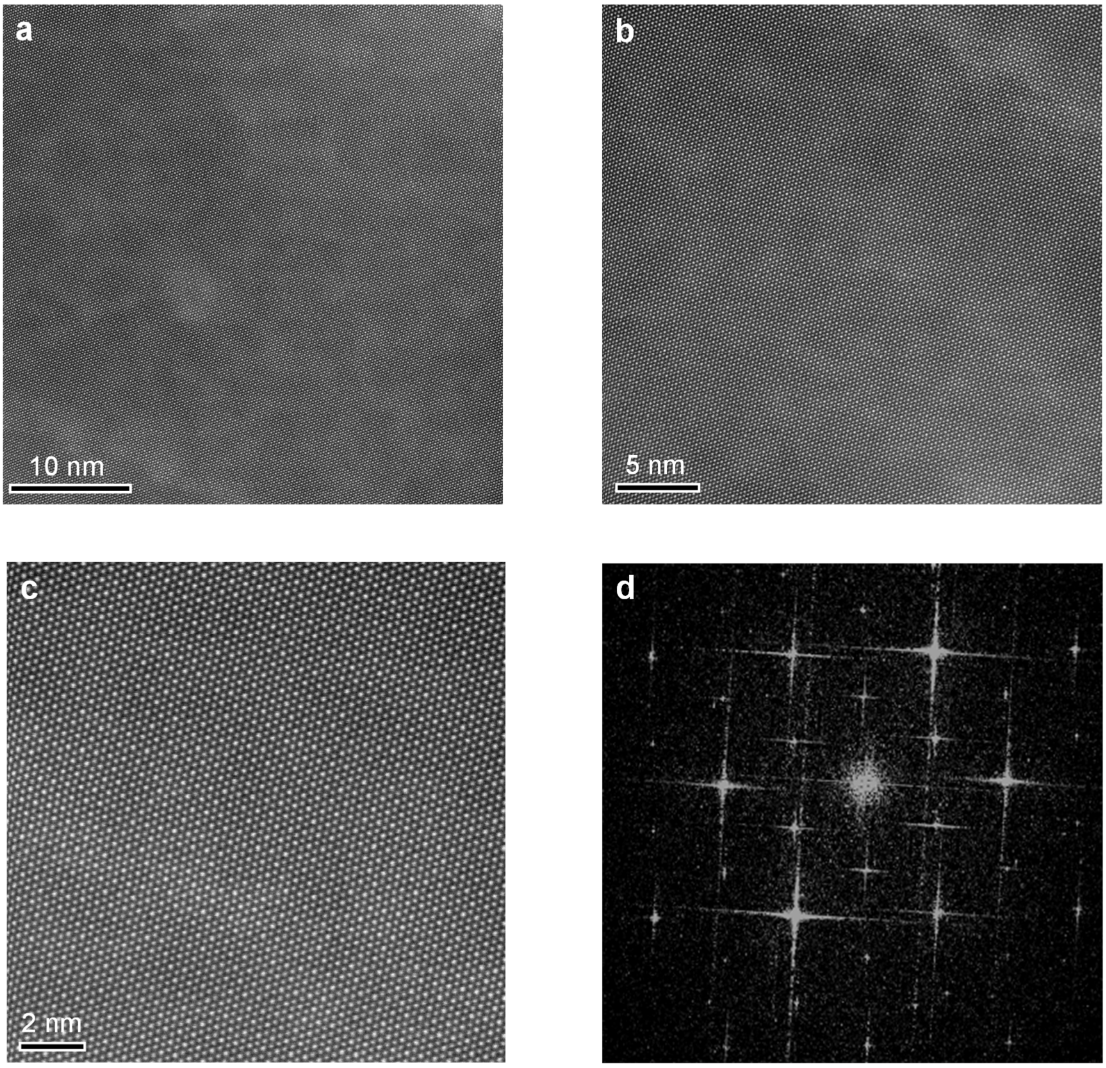

Supplementary Figure 7. STEM-HAADF images for the top view of the $PtTe_2$ thin films. a-c, HR-STEM images with the different magnification. **d**, The corresponding fast Fourier transform pattern taken from the whole region of **a**. The distinct hexagonal symmetry in the atomic structure reflects the 1*T*-phase structure.

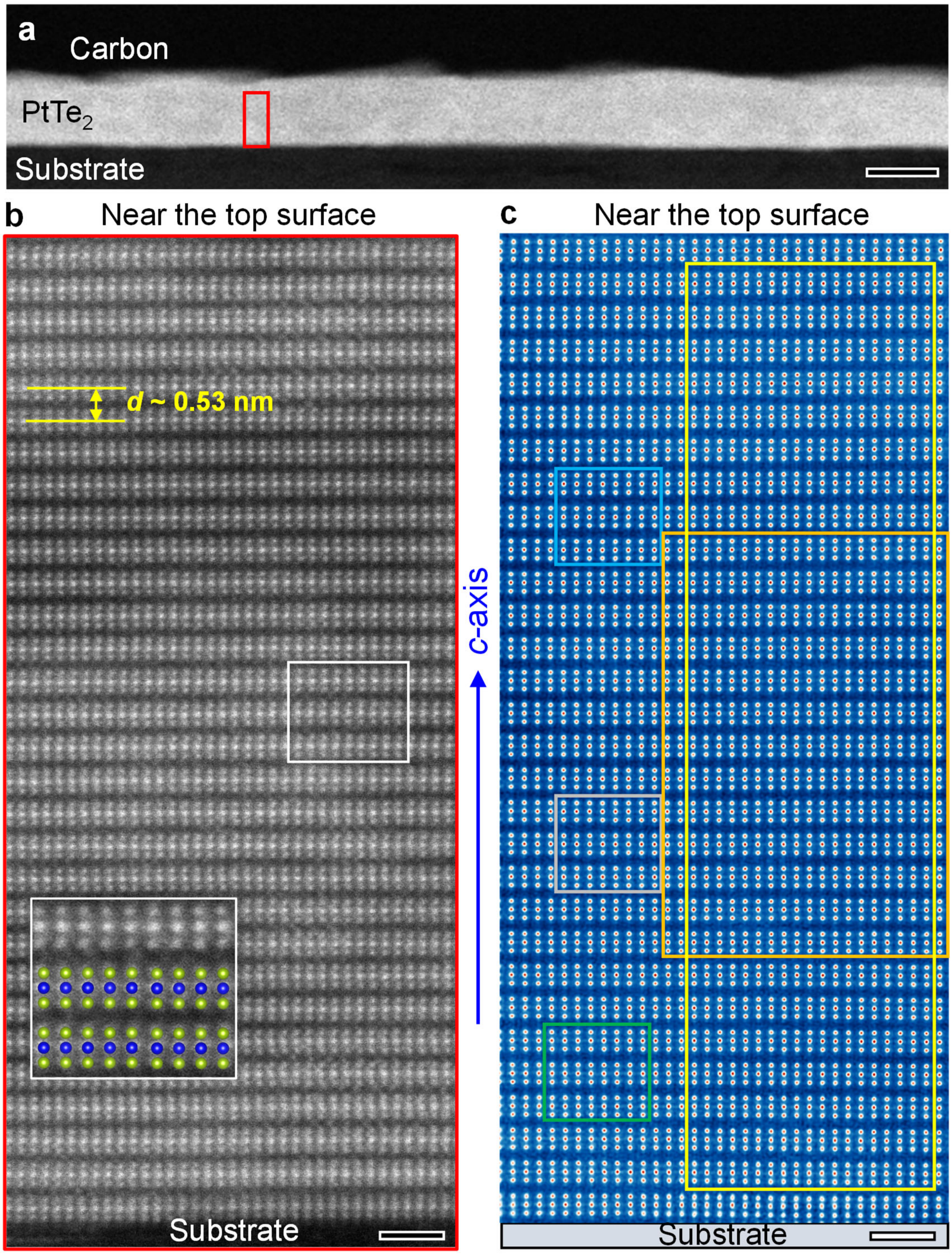

Supplementary Figure 8. The cross-sectional images of $PtTe_2$ film with the thickness of 18 nm *via* different imaging techniques. a, The low-magnified STEM-HAADF image. The scale bar is 20 nm. **b**, The high-resolution STEM-HAADF image taken from the boxed area in **a**. The inset shows a magnified view of the white boxed area in **b**. The scale bar is 1 nm. **c**, The corresponding total phase image summed over all 15 slices reconstructed with the electron ptychography. The substrate is schematically shown. The scale bar is 1 nm. The images taken from orange, blue, grey, and green rectangles are shown in Fig. 1b,c of the main text, respectively.

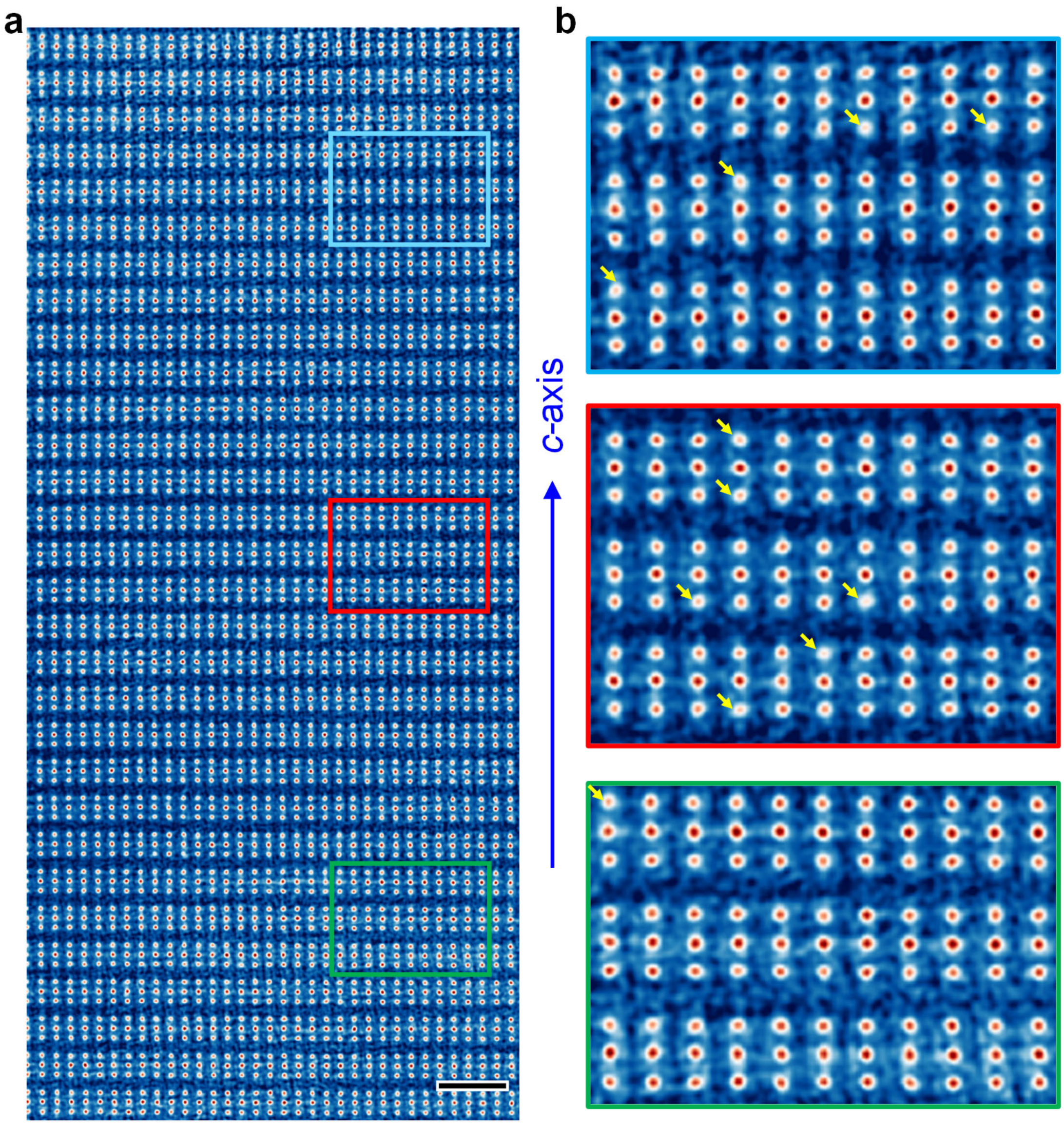

Supplementary Figure 9. The phase image of a slice reconstructed with electron ptychography. a, The phase images of [001] $PtTe_2$ of one slice. The scale bar is 1 nm. **b**, The corresponding phase images taken from blue, red, and green rectangles in **a**, respectively. The yellow arrows denote the V_{Te} due to a relatively weak colour contrast.

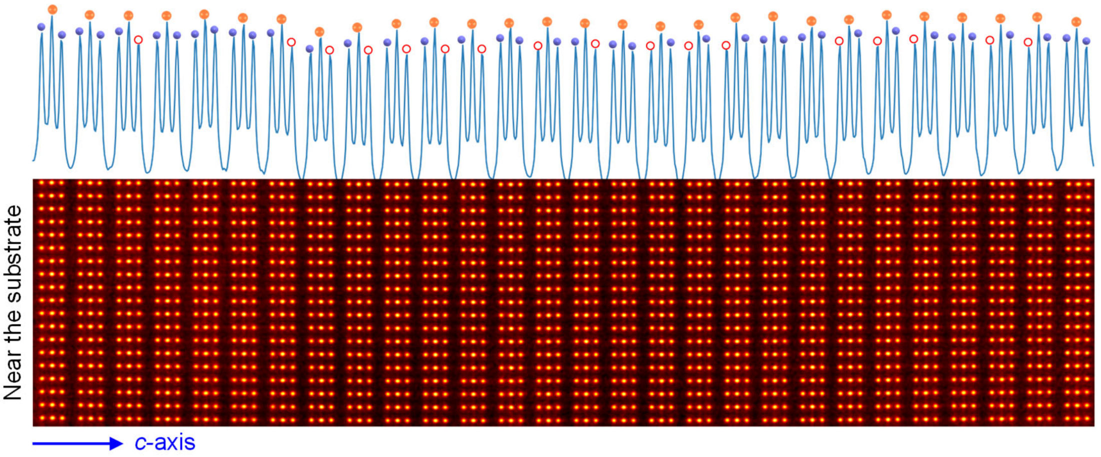

Supplementary Figure 10. The profile of phase intensity along the yellow rectangle in Supplementary Fig. 8c. The strongest peaks come from Pt atoms, while the peaks on either side come from Te atoms. The weaker Te peaks represent the presence of V_{Te} in the Te atomic columns.

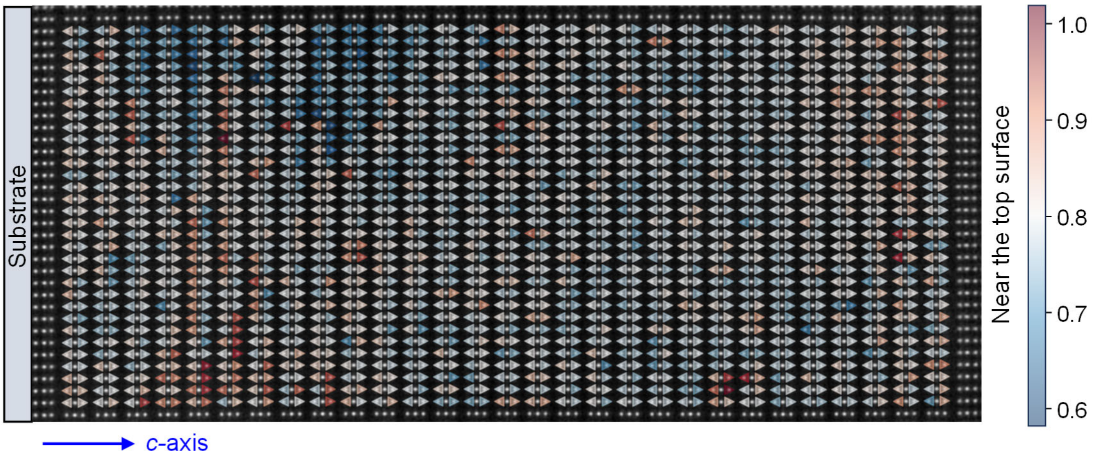

Supplementary Figure 11. The relative phase mapping of the Te/Pt in the selected 29-layer $PtTe_2$ film from near the substrate to near the top surface. Blue and red colours denote the high and low V_{Te} concentration, respectively. It can be deduced that the V_{Te} defect is concentrated in the midst of films, in good agreement with the Te phase mapping results shown in Fig. 1e. The substrate is schematically shown. Figure 1f is extracted from this figure.

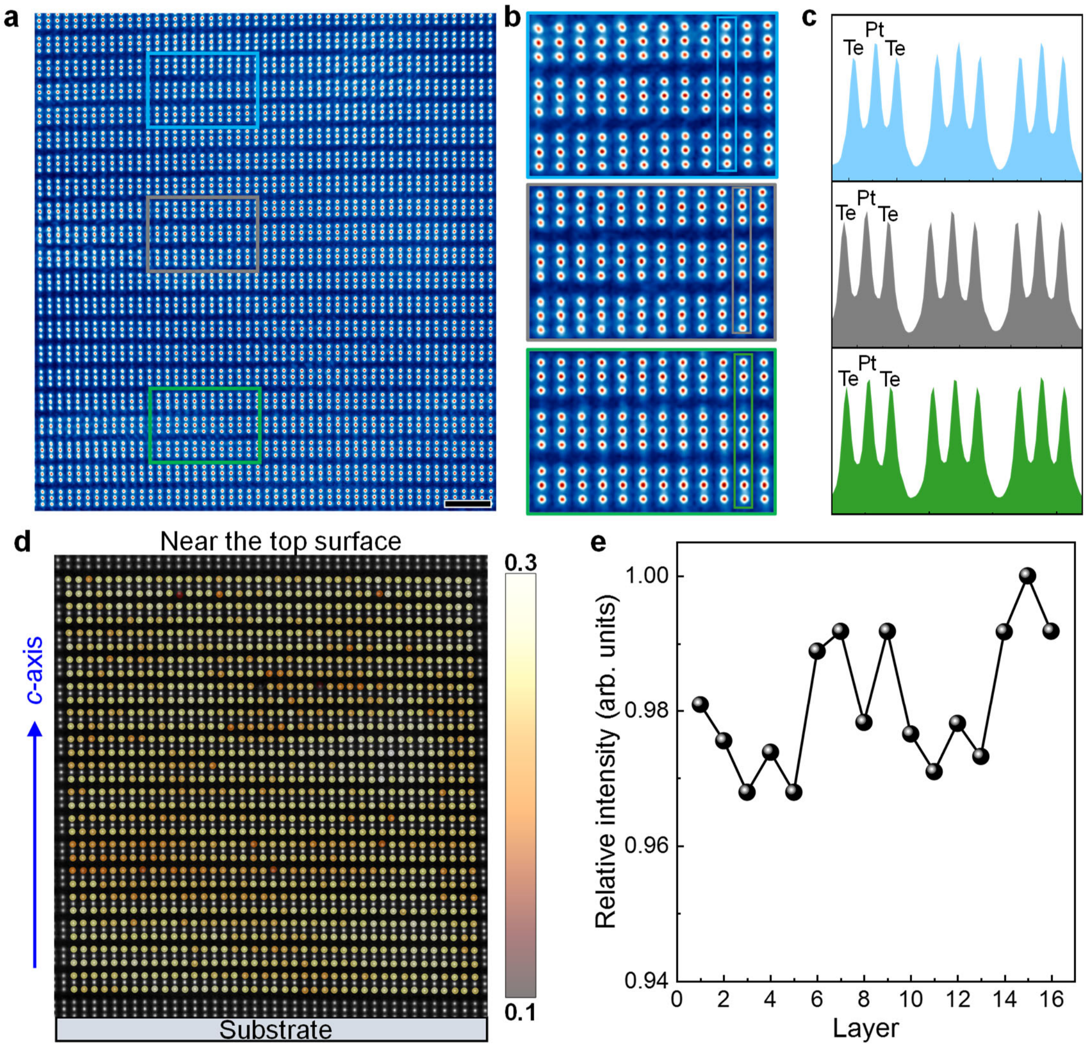

Supplementary Figure 12. The multislice electron ptychography for depth sectioning of a Te-passivated $PtTe_2$ sample. a, The corresponding total phase image summed over all 15 slices reconstructed with the electron ptychography. The scale bar is 1 nm. **b**, The phase images marked with blue, grey and green rectangles taken from the top, middle and bottom regions in **a**, respectively. **c**, The corresponding profiles of phase intensity taken from atomic columns marked with blue, grey and green rectangles in **b**, respectively. **d**, Te phase mapping in the selected 16-layer $PtTe_2$ film from near the substrate to near the top surface. Light and dark colours denote the low and high V_{Te} concentration, respectively. The colorbar denotes the Te phase intensity with the unit of rad. The substrate is also schematically shown. **e**, The relative phase intensity variation of the Te/Pt in each $PtTe_2$ layer. The intensity is normalized by maximum intensity. These results are distinctly different from those of V_{Te}-gradient $PtTe_2$ films shown in Fig. 1, indicative of the negligible V_{Te}.

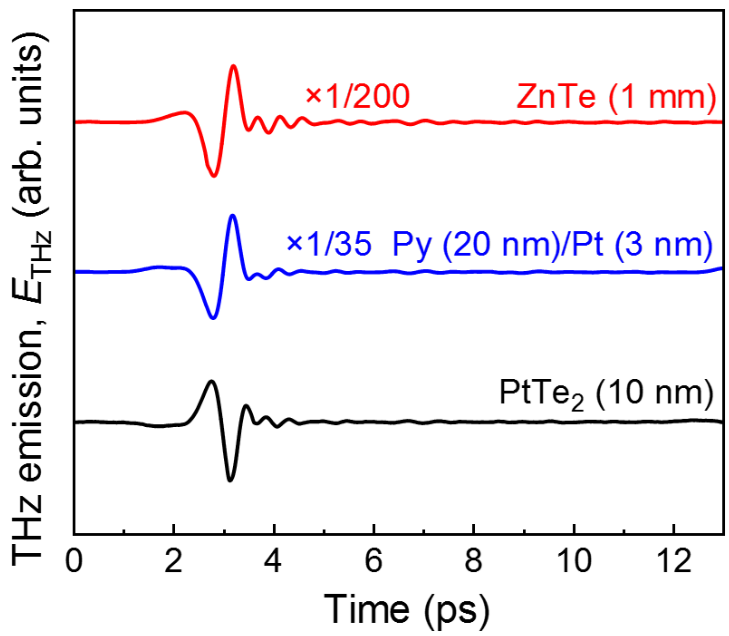

Supplementary Figure 13. THz emission from 1-mm-thick ZnTe crystal, Py (20 nm)/Pt (3 nm) heterostructures, and 10-nm-thick $PtTe_2$ films under the linearly polarized laser excitation of 160 μJ cm^{-2}. The THz emission amplitudes of ZnTe and Py/Pt are divided by 200 and 35 times for comparison, respectively.

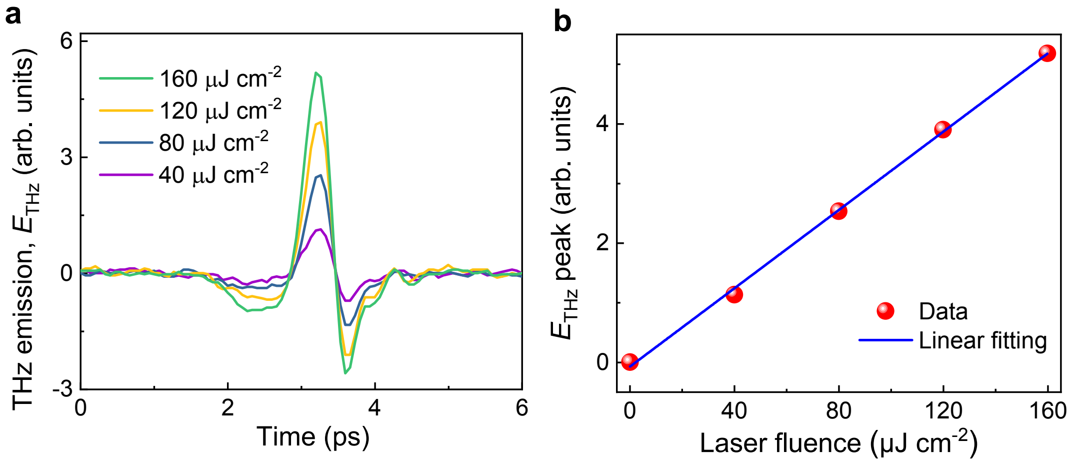

Supplementary Figure 14. Pump fluence dependence of the THz emission under the linear polarized excitation of the as-grown $PtTe_2$ films with the thickness of 10 nm. a, Transient THz waveforms under different pump fluences. **b**, The extracted THz peak amplitude versus the laser fluence. The THz amplitude increases linearly with increasing pump fluence, indicating that the emitted THz signals are dominated by a second-order nonlinear effect.

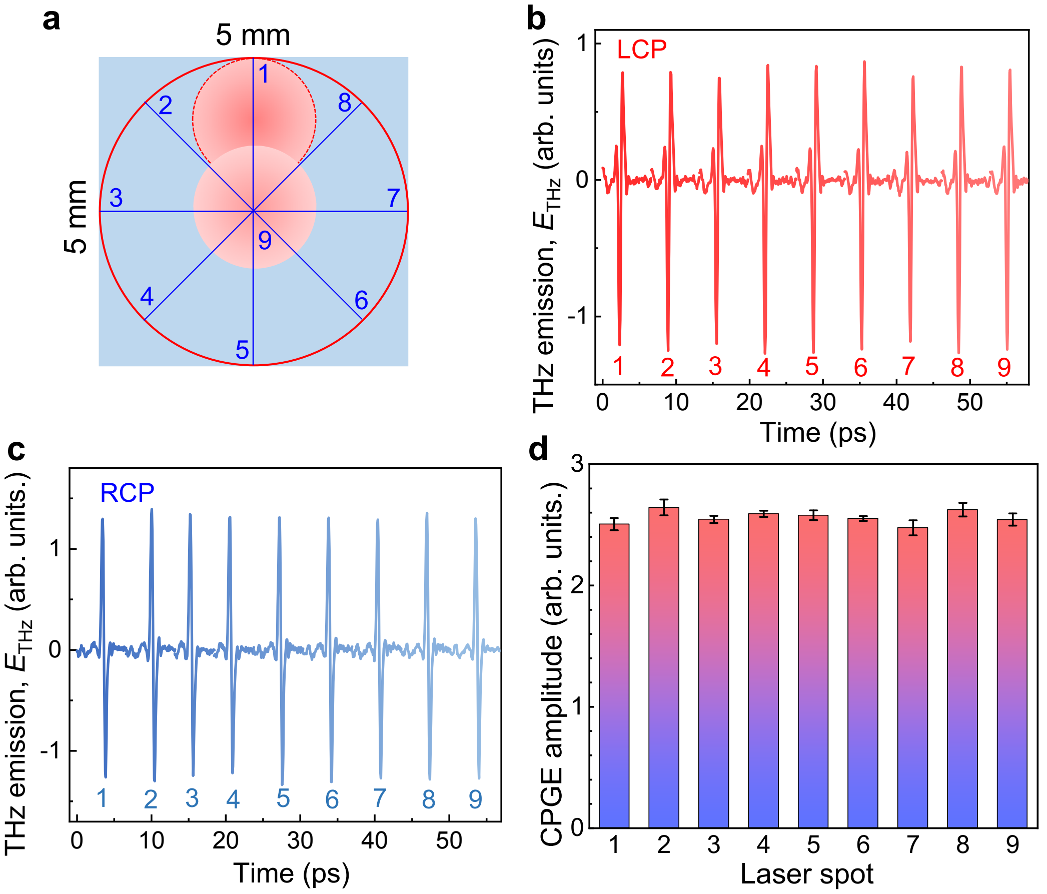

Supplementary Figure 15. Helicity dependent THz emission acquired from various areas in a single $PtTe_2$ film with the thickness of 10 nm. a, Schematic of THz emission measurements with the pump beam hitting different areas, where the blue rectangle represents the $PtTe_2$ film with the size of 5×5 mm^2 and the red circle represents the laser spot with a diameter of about 2 mm. The THz emission measurements of the other 8 regions surrounding the spot "9" are also measured, with the spot "9" at the center. **b**,**c**, Transient THz waveforms under the LCP and RCP excitations at $\varphi = 0^o$, respectively. **d**, The CPGE amplitude with subtraction of $E_{LCP}-E_{RCP}$ from **b** and **c**, respectively. The obtained almost constant CPGE amplitude confirms the in-plane homogeneity of films. The error bars indicate the uncertainty from the experimental results.

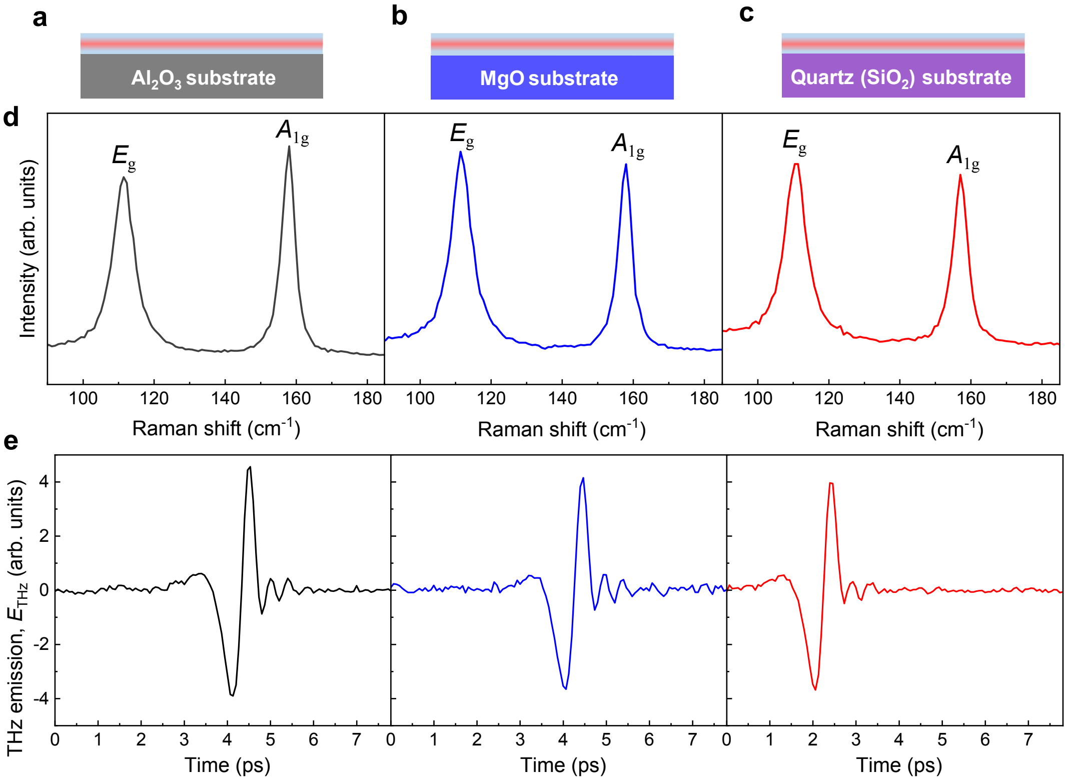

Supplementary Figure 16. Raman spectra and THz emission of 18-nm-thick $PtTe_2$ films grown on different substrates. a-c, The schematics of the defect-gradient $PtTe_2$ films grown on (0001)-Al_2O_3, (111)-MgO and quartz glass (SiO_2) substrates, respectively. **d,e**, Raman spectra and THz emission of $PtTe_2$ films grown on different substrates, respectively. The THz emission measurements are conducted under the linearly polarized excitation with the sample azimuth angle of zero (i.e., φ = 0°).

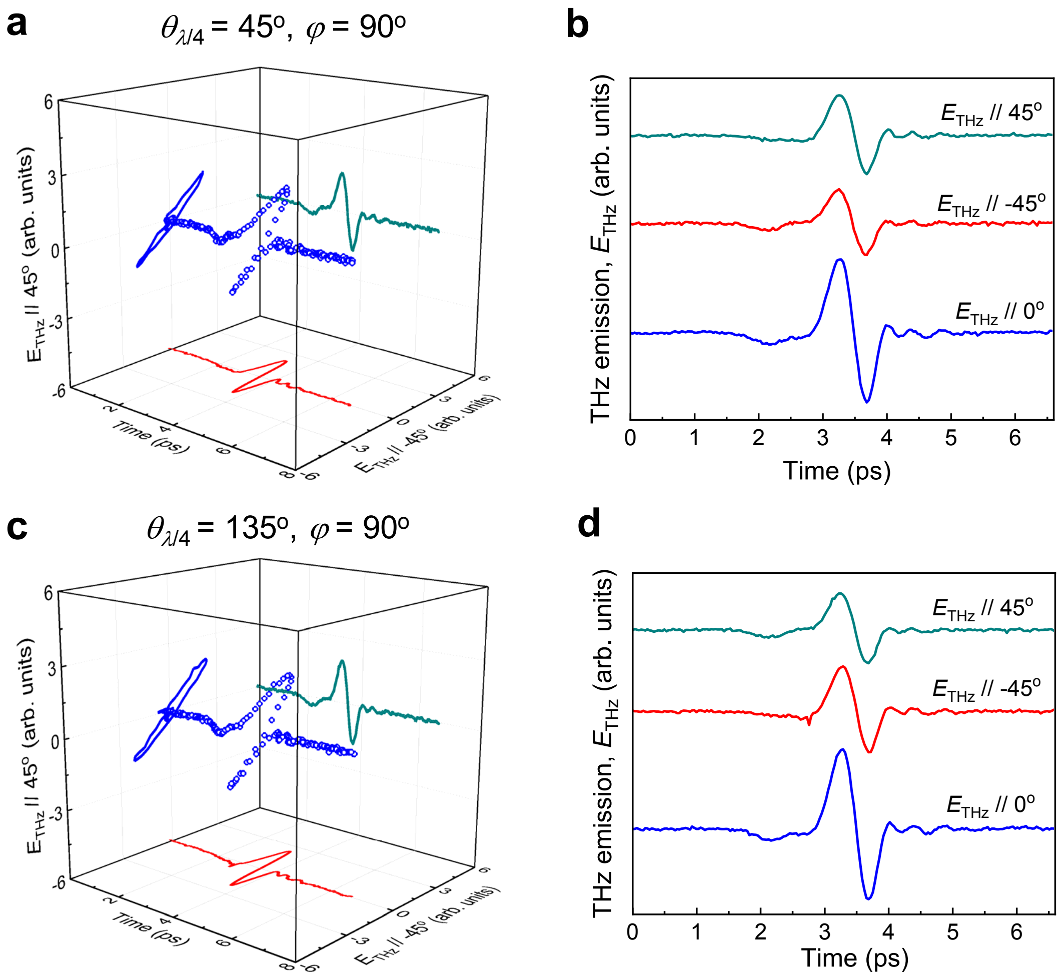

Supplementary Figure 17. The polarization state of the emitted THz from $PtTe_2$ films with the thickness of 10 nm. a,c, Three-dimensional trajectory plots of temporal waveforms of the THz emission for the sample azimuth angle $\varphi = 90^{o}$ under the LCP ($\theta_{\lambda/4} = 45^{o}$) and RCP ($\theta_{\lambda/4} = 135^{o}$), respectively. **b,d**, The corresponding THz waveforms of **a** and **c**, respectively.

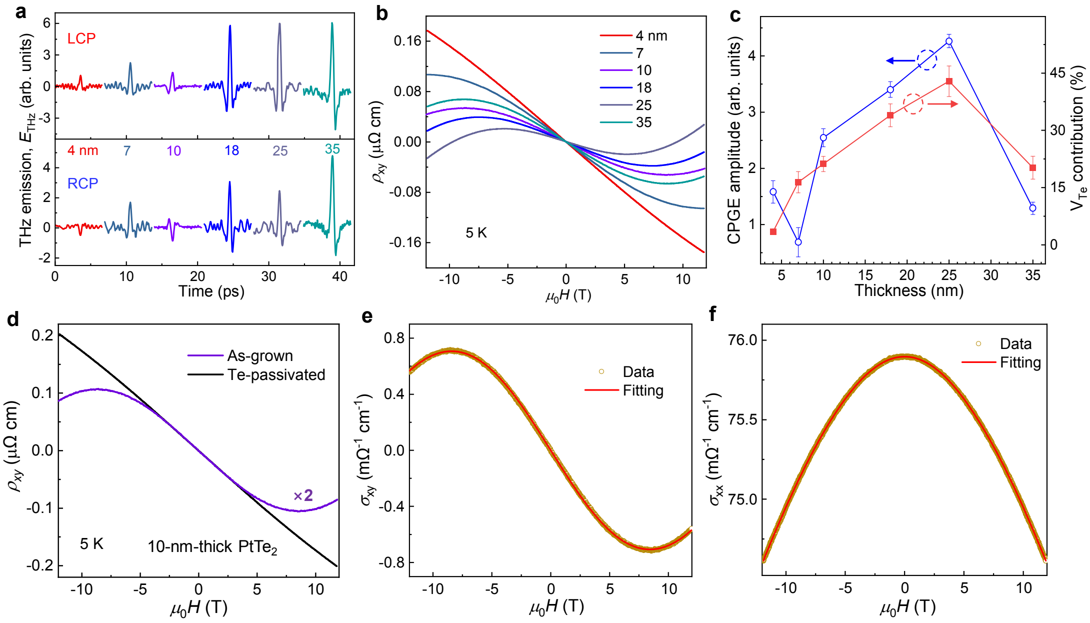

Supplementary Figure 18. Thickness-dependent THz emission, CPGE amplitude and V_{Te} contribution of $PtTe_2$ films. a, The transient THz waveforms with different thicknesses under LCP and RCP at φ = 0°, respectively. **b**, The Hall curves with different $PtTe_2$ thicknesses under the perpendicular measurement geometry at 5 K. **c**, The extracted CPGE amplitude from **a** and V_{Te} contribution from **b** as a function of thickness. The lines are drawn as guides for the eyes. The error bars of the CPGE amplitude and the V_{Te} contribution indicate the uncertainties from the calculations of the difference between LCP and RCP and the fitting of the experimental results, respectively. **d**, The Hall behaviors of the as-grown and passivated $PtTe_2$ films at 5 K. **e,f**, Field-dependent transverse and longitudinal conductivities of the as-grown 10-nm-thick sample, which are fitted by the two-band model. For details refer to Supplementary Note 5.

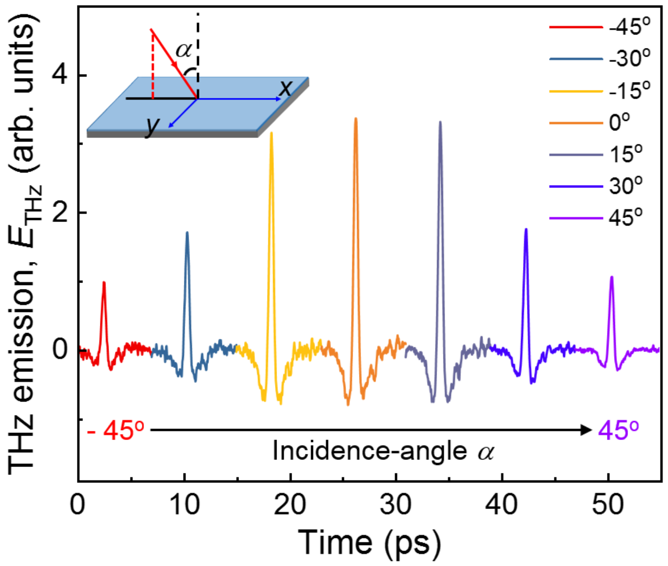

Supplementary Figure 19. Incidence-angle-dependent THz emission from 35-nm-thick $PtTe_2$ films under the linearly polarized excitation. The emitted THz polarity does not show the reversal behavior at the negative and positive incident angles, indicating that the PDE process is unlikely the radiation mechanism. The inset is the schematic of THz emission with the different incident angles (α).

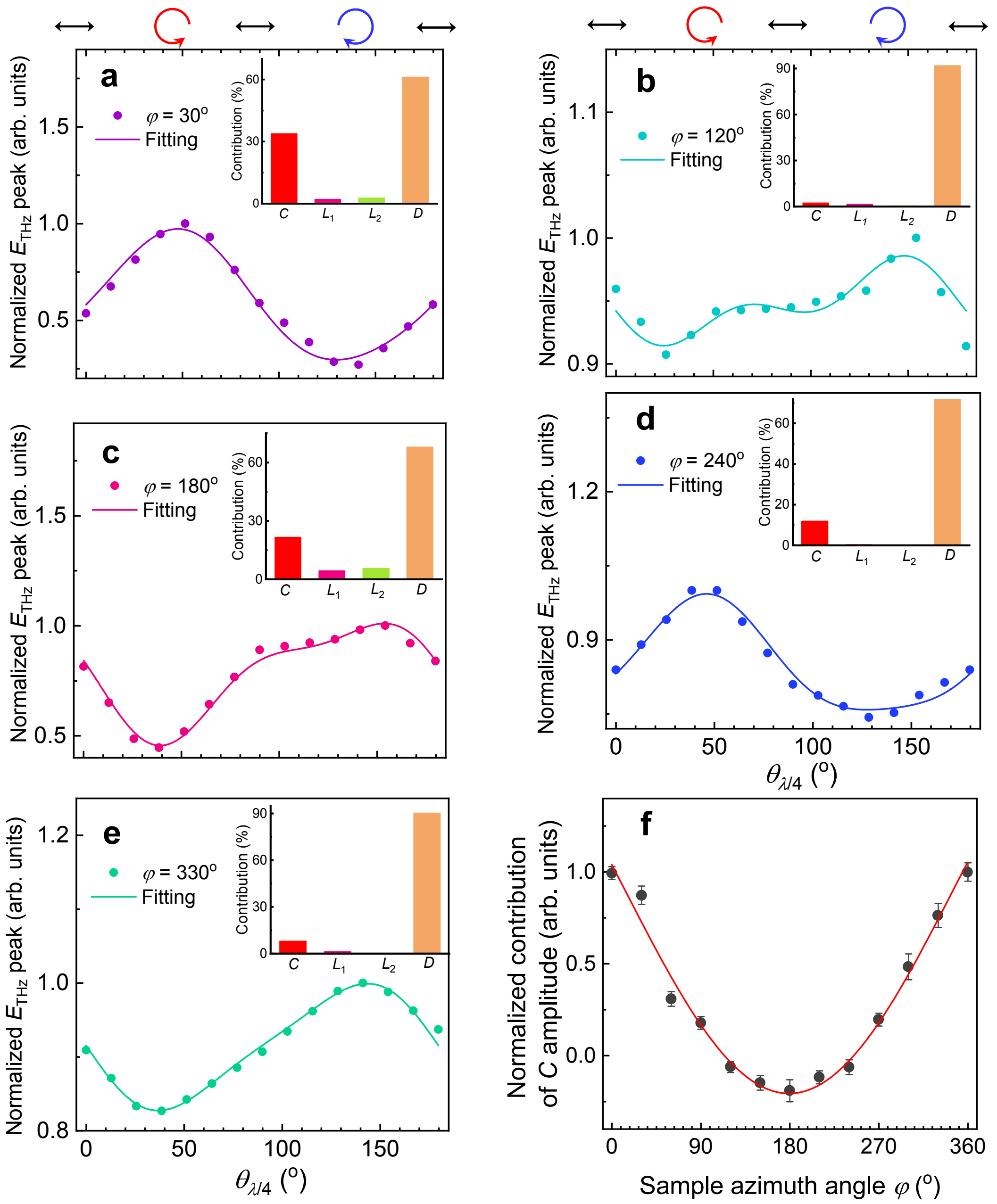

Supplementary Figure 20. The CPGE contribution (*C* term) as a function of the sample azimuthal angle *φ*. a-e, Part of the THz peak amplitudes with different φ as a function of $\theta_{\lambda/4}$, where the solid lines represent the fitting results. The insets show the normalized fitting parameters extracted from the THz emission. **f**, The extracted C term as a function of φ. The solid curves in **a-e** are fitted by $J = C\sin 2\theta + L_1 \sin 4\theta + L_2 \cos 4\theta + D$. The solid curve in **f** is fitted by cosφ.

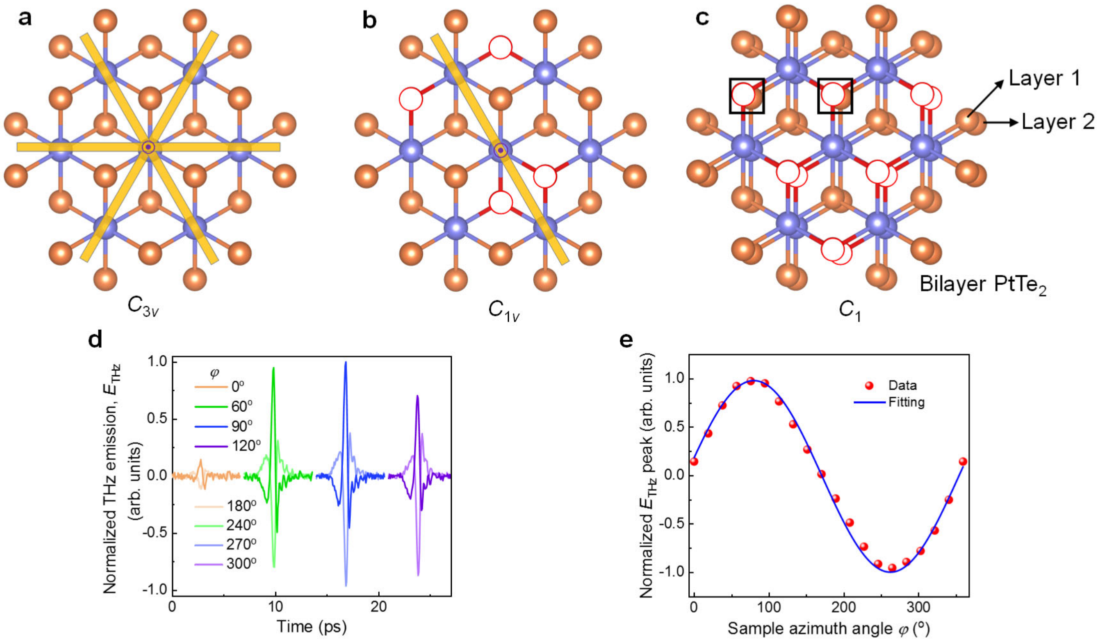

Supplementary Figure 21. In-plane symmetry analysis of the $PtTe_2$ films. a-c, Schematic diagrams (top view) of the crystal structures of $PtTe_2$ films without and with the V_{Te} defect, corresponding to the C_{3v} symmetry (**a**), the C_{1v} symmetry (**b**) and C_1 symmetry (**c**), respectively. The V_{Te} defect in **b** is generated symmetrically under a mirror-plane symmetry axis, which is extremely difficult to control during the preparation. The orange strips represent the vertical mirror planes. Circled dots represent the rotational axis. Here, only monolayer and bilayer crystal structures for $PtTe_2$ are illustrated for simplicity to clearly show the change of the point group. The orange boxed region in **c** indicates that Layer 1 has a V_{Te} defect in this region that is absent in Layer 2. Note that Layer 1 with the V_{Te} defect can still maintain the C_{3v} symmetry due to the relaxation of the atoms. Nevertheless, because of the different V_{Te} defect concentrations between the neighboring layers, the overall symmetry can be still reduced to C_1. Note that we are focused only on the rotational symmetries around the z axis and the vertical mirror symmetry. **d**, Transient THz waveforms at various sample azimuthal angle (φ). **e**, The extracted THz peak amplitude under linear polarized excitation as a function of the φ from **d**. The solid line is fitted by cosφ, indicating the C_1 symmetry. For details refer to Supplementary Note 4.

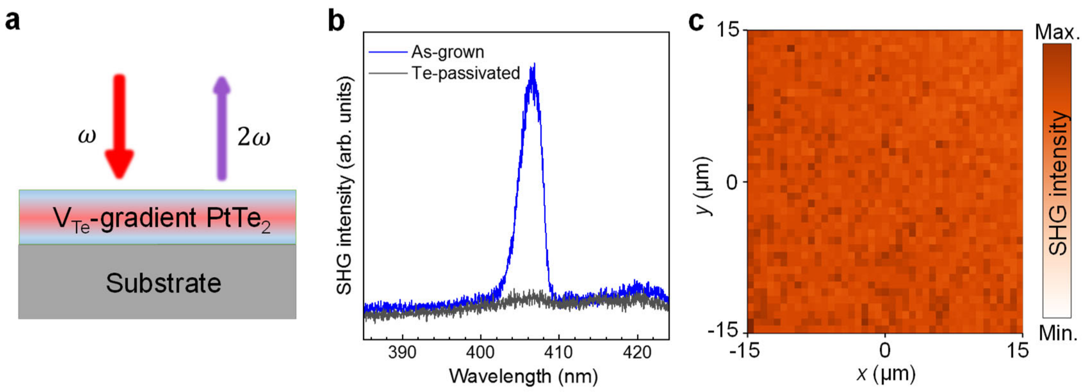

Supplementary Figure 22. Second harmonic generation (SHG) of the 18-nm-thick $PtTe_2$ films. a, Schematic of the emergence of the SHG in the V_{Te}-gradient $PtTe_2$ films. Excitation at ω (red arrow) generates second-harmonic radiation at 2ω (purple arrow). **b**, Frequency-up-converted optical spectra at the excitation wavelength of 810 nm for the as-grown $PtTe_2$ films and Te-passivated $PtTe_2$ films, respectively. **c**, The SHG mapping for the as-grown $PtTe_2$ films with a region of 30 × 30 μm^2. A uniform colour in the mapping images indicates that symmetry breaking occurs on a large scale, not limited in a locally small region.

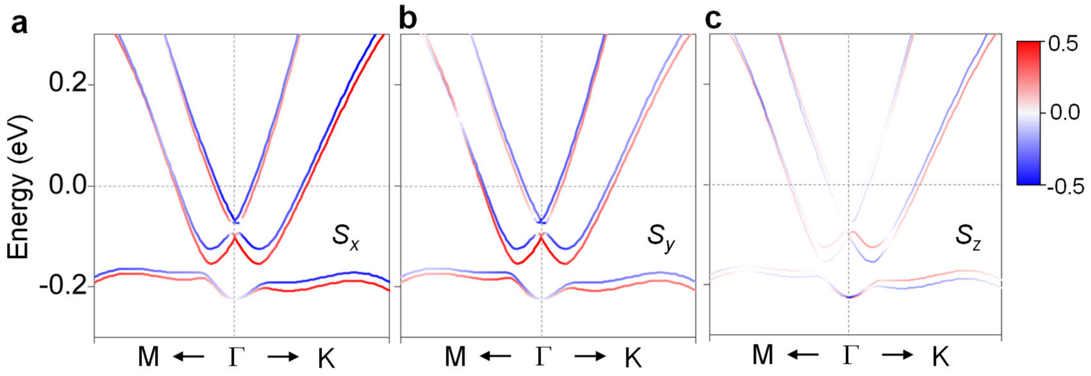

Supplementary Figure 23. Band structures near the Fermi energy for the bulk samples with a defect gradient. a-c, The average spin components along the *x*, *y*, and *z* directions are marked by colour. It is the supplementary information of Fig. 3c in the main text.

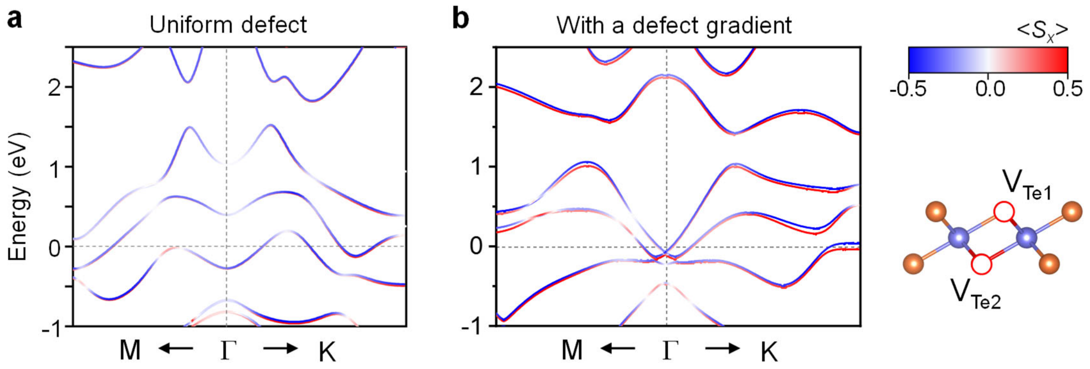

Supplementary Figure 24. The comparison of the band structures of the $PtTe_2$ with the uniform defect (a) and a gradient-like defect (b) along the depth direction. It can be seen that only the vertical (out-of-plane) defect gradient can create the sizable band spin splitting, resulting in the increased subbands.

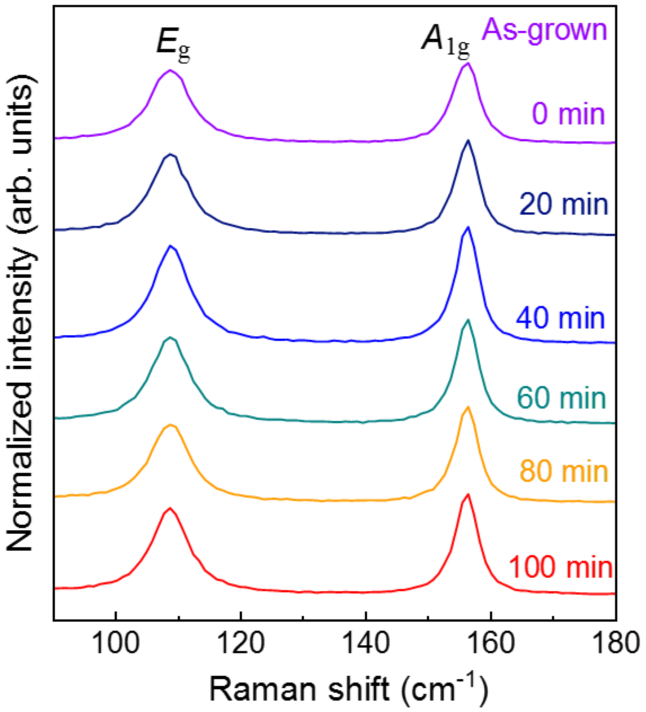

Supplementary Figure 25. Raman spectra of the 18-nm-thick $PtTe_2$ films with various *in-vacuo* annealing time. There are no obvious vibrational peaks' shifts and intensity changes for films under the various annealing time, indicating the negligible structural changes of films during *in-vacuo* annealing.

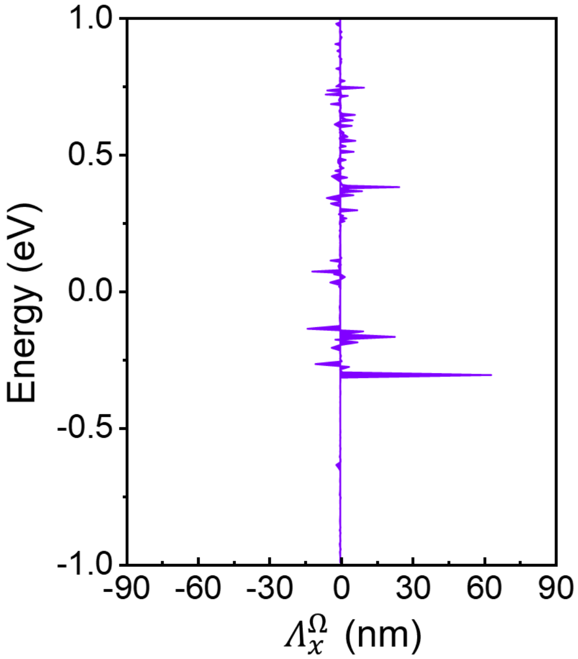

Supplementary Figure 26. The *x* component of BCD (Λ_x^{Ω}) for the defective $PtTe_2$ sample. The BCD value can reach the order of 10 nm, with a maximum value up to ~64 nm. It is the broader BCD range for the right panel in Fig. 4f.

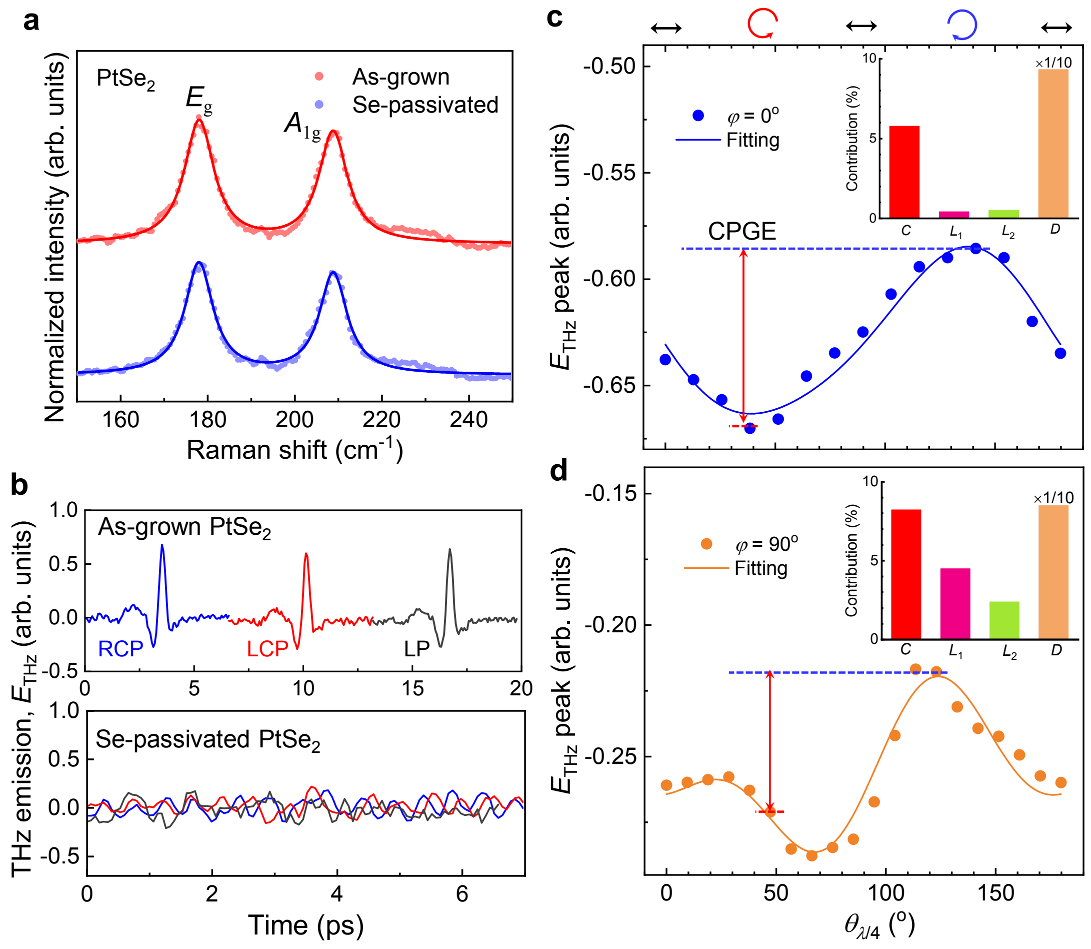

Supplementary Figure 27. Helicity dependent THz emission in 10-nm-thick Dirac semimetal $PtSe_2$ thin films containing the Se-vacancy defect gradient. a, Raman spectra of the as-grown and Se-passivated $PtSe_2$ films. **b**, Transient THz waveforms from the as-grown $PtSe_2$ films (top panel) and passivated $PtSe_2$ films (bottom panel) under the RCP, LCP and LP excitations at $\varphi = 0^{\circ}$, respectively. **c**,**d**, THz peak amplitudes at $\varphi = 0^{\circ}$ (blue plot) and $\varphi = 90^{\circ}$ (orange plot) as functions of $\theta_{\lambda/4}$, where the solid lines represent the fitting. The insets show the normalized fitting parameters extracted from the THz emission. The THz emission performance is also summarized in Supplementary Table 1. The data further strengthen our universal film-growth method as well as the unique strategy of the symmetry engineering, thus enabling the observation of the helicity dependent THz emission in centrosymmetric Dirac semimetals.

Supplementary Tables

Supplementary Table 1. Summarized THz amplitudes of typical materials and their THz emission efficiency (η). Here, E_0 is the THz peak with the unit of V cm^{-1}, F is the pump fluence, d is the penetration depth for 800-nm light or the thickness of the sample, and η is the THz emission efficiency per length [$\eta = E_0/(Fd)$].

Material	E_0 (t) (V cm^{-1})	F (μJ cm^{-2})	d (nm)	η (V J^{-1})	Reference
ZnTe	687	160	1×10^6	4.29×10^7	This work
Py/Pt	122	160	20/3	3.31×10^{11}	This work
Defect-gradient $PtTe_2$	3.58−16.77	160	10−35	2.24×10^{10} – 3.00×10^{10}	This work
Defect-gradient $PtSe_2$	0.67	160	10	4.19×10^9	This work
$PtSe_2$	23.5	283	16	5.2×10^{10}	ref. 37
TaAs	~600	2830	25	8.5×10^{10}	ref. 38

Note: The THz amplitude of $PtSe_2$ sample in ref. 37 is nearly zero when the measurement condition is the same as ours (i.e., pump under normal incidence).

Supplementary Table 2. The fitting carrier concentration and mobility using the classical two-band model for the as-grown $PtTe_2$ films with the different thickness at 5 K. These are the relevant data for Supplementary Fig. 18b,c.

Thickness (nm)	n_e ($\times 10^{18}$ cm^{-3})	μ_e (cm^{2}V^{-1}s^{-1})	n_h ($\times 10^{18}$ cm^{-3})	μ_h (cm^{2}V^{-1}s^{-1})	V_{Te} contribution (%)
4	6.72	2620.32	6.72	94.76	3.49
7	7.92	1810.64	7.92	355.46	16.41
10	11.62	1430.21	11.62	384.55	21.19
18	14.29	1143.53	14.29	587.26	33.93
25	18.68	925.23	18.68	694.29	42.87
35	9.51	1590.41	9.51	403.23	20.22

Supplementary Table 3. Summarized spin splitting energy (*Δ*) of the typical materials.

	Material	*Δ* (meV)	Reference
Dirac or Weyl semimetal	$PtTe_2$ films with the vertical V_{Te} defect gradient	15±5 (Γ point)<br>70±5 (K point)	This work
	TaAs	Not shown	ref. 38
	CoSi	20	ref. 39
	RhSi	Not shown	ref. 40
	WTe_2	15–25	ref. 41
	Monolayer WTe_2	<30	ref. 31
Semiconductor	$(4,4\text{-DFPD})_2PbI_4$	60	ref. 42
	(2D)-phenethylammonium lead iodide	35±10	ref. 43
	2D-OIHP lead(II) iodide	<50	ref. 44
	$(C_6H_5C_2H_4NH_3)_2PbI_4$	40±5	ref. 45
	BiTeI	100	ref. 46
	α-GeTe	190	ref. 47
	Monolayer $PtSe_2$ with defects	152 (K point)	ref. 48
	Gated monolayer $PtSe_2$	0.1–50	ref. 49
	Monolayer $MoSe_2$	180	ref. 50
	$(NH_2CHNH_2)SnI_3$	10–15	ref. 51
Rashba system	Bi_2Se_3	180	ref. 52
	$Al_{0.25}Ga_{0.75}N/GaN$	9	ref. 53
	$LaAlO_3/SrTiO_3$	<10	ref. 54
	$LaAlO_3/KTaO_3$	19.02−25.42	ref. 55
	$In_{0.53}Ga_{0.47}As/In_{0.52}Al_{0.48}As$ quantum well	4.5−6	ref. 56
	InAs/AlSb quantum well	1.5−3.5	ref. 57
	GaSb/InAs/GaSb quantum well	3.7	ref. 58

Note: The green-shaded rows stand for the observation of the both helicity dependent THz emission (also CPGE) and spin splitting. The green and yellow-shaded rows stand for the observation of the both CPGE and spin splitting. While the blank ones stand for the observation of the only spin splitting without the CPGE.

Supplementary Table 4. The fitting carrier concentration and mobility using the classical two-band model for 18-nm-thick $PtTe_2$ films with various *in-vacuo* annealing time at 5 K. These are the relevant data for Fig. 4b,c.

***In-vacuo* annealing time (min)**	**n_e ($\times 10^{18}$ cm^{-3})**	**μ_e (cm^{2}V^{-1}s^{-1})**	**n_h ($\times 10^{18}$ cm^{-3})**	**μ_h (cm^{2}V^{-1}s^{-1})**	**V_{Te} contribution (%)**
0	10.23	1565.31	10.23	327.68	17.31
20	11.28	1423.46	11.28	360.55	20.21
40	15.74	1058.20	15.74	480.23	31.25
60	17.86	475.62	17.86	546.84	38.46
80	15.26	1061.23	15.26	478.25	31.07
100	14.13	939.57	14.13	308.53	24.72

Supplementary References

1. Lin, H. *et al.* Growth of environmentally stable transition metal selenide films. *Nat. Mater.* **18**, 602-607 (2019).
2. Xu, H. *et al.* High spin Hall conductivity in large-area type-II Dirac semimetal $PtTe_2$. *Adv. Mater.* **32**, 2000513 (2020).
3. Suo, P. *et al.* Observation of negative terahertz photoconductivity in large area type-II Dirac semimetal $PtTe_2$. *Phys. Rev. Lett.* **126**, 227402 (2021).
4. Li, X. *et al.* Ordered clustering of single atomic Te vacancies in atomically thin $PtTe_2$ promotes hydrogen evolution catalysis. *Nat. Commun.* **12**, 2351 (2021).
5. Fang, H. *et al.* Atomically precise vacancy-assembled quantum antidots. *Nat. Nanotechnol.* **18**, 1401-1408 (2023).
6. Kong, D. *et al.* Synthesis of MoS_2 and $MoSe_2$ films with vertically aligned layers. *Nano Lett.* **13**, 1341-1347 (2013).
7. Malekpour, H. & Balandin, A. A. Raman-based technique for measuring thermal conductivity of graphene and related materials. *J. Raman Spectrosc.* **49**, 106-120 (2018).
8. Parkin, W. M. *et al.* Raman shifts in electron-irradiated monolayer MoS_2. *ACS Nano* **10**, 4134-4142 (2016).
9. Cote, D., Laman, N. & van Driel, H. M. Rectification and shift currents in GaAs. *Appl. Phys. Lett.* **80**, 905-907 (2002).
10. Obraztsov, P. A. *et al.* Photon-drag-induced terahertz emission from graphene. *Phys. Rev. B* **90**, 241416(R) (2014).
11. Shalygin, V. A., Moldavskaya, M. D., Danilov, S. N., Farbshtein, I. I. & Golub, L. E. Circular photon drag effect in bulk tellurium. *Phys. Rev. B* **93**, 045207 (2016).
12. Jiang, C. *et al.* Helicity dependent photocurrents in graphene layers excited by midinfrared radiation of a CO_2 laser. *Phys. Rev. B* **84**, 125429 (2011).
13. Strecker, K. E., Partridge, G. B., Truscott, A. G. & Hulet, R. G. Formation and propagation of matter-wave soliton trains. *Nature* **417**, 150-153 (2002).
14. Benicewicz, P. K., Roberts, J. P. & Taylor, A. J. Scaling of terahertz radiation from large-aperture biased photoconductors. *J. Opt. Soc. Am. B* **11**, 2533-2546 (1994).
15. Pettine, J. *et al.* Ultrafast terahertz emission from emerging symmetry-broken materials. *Light Sci. Appl.* **12**, 133 (2023).
16. Sipe, J. E. & Shkrebtii, A. I. Second-order optical response in semiconductors. *Phys. Rev. B* **61**, 5337-5352 (2000).
17. Ganichev, S. D. & Prettl, W. Spin photocurrents in quantum wells. *J. Phys.: Condens. Matter* **15**, R935-R983 (2003).
18. Le, C. & Sun, Y. Topology and symmetry of circular photogalvanic effect in the chiral multifold semimetals: a review. *J. Phys. Condens. Matter* **33**, 503003 (2021).
19. Quereda, J., Ghiasi, T. S., You, J.-S., van den Brink, J., van Wees, B. J. & van der Wal, C. H. Symmetry regimes for circular photocurrents in monolayer

$MoSe_2$. *Nat. Commun.* **9**, 3346 (2018).
20. Absor, M. A. U. *et al.* Defect-induced large spin-orbit splitting in monolayer $PtSe_2$. *Phys. Rev. B* **96**, 115128 (2017).
21. Zheng, Z. *et al.* Field-free spin-orbit torque-induced switching of perpendicular magnetization in a ferrimagnetic layer with a vertical composition gradient. *Nat. Commun.* **12**, 4555 (2021).
22. Liu, L. *et al.* Current-induced self-switching of perpendicular magnetization in CoPt single layer. *Nat. Commun.* **13**, 3539 (2022).
23. Xie, X. *et al.* Controllable field-free switching of perpendicular magnetization through bulk spin-orbit torque in symmetry-broken ferromagnetic films. *Nat. Commun.* **12**, 2473 (2021).
24. Huang, Q. *et al.* Field-free magnetization switching in a ferromagnetic single layer through multiple inversion asymmetry engineering. *ACS Nano* **16**, 12462-12470 (2022).
25. Liang, J., Chshiev, M., Fert, A. & Yang, H. Gradient-induced Dzyaloshinskii–Moriya interaction. *Nano Lett.* **22**, 10128-10133 (2022).
26. Chen, L. *et al.* Engineering symmetry breaking enables efficient bulk spin-orbit torque-driven perpendicular magnetization switching. *Adv. Funct. Mater.* **33**, 2308823 (2023).
27. Duan, S. *et al.* Berry curvature dipole generation and helicity-to-spin conversion at symmetry-mismatched heterointerfaces. *Nat. Nanotechnol.* **18**, 867-874 (2023).
28. Ma, Q. *et al.* Observation of the nonlinear Hall effect under time-reversal-symmetric conditions. *Nature* **565**, 337-342 (2019).
29. Du, L. *et al.* Engineering symmetry breaking in 2D layered materials. *Nat. Rev. Phys.* **3**, 193-206 (2021).
30. Yu, X. *et al.* Atomically thin noble metal dichalcogenide: a broadband mid-infrared semiconductor. *Nat. Commun.* **9**, 1545 (2018).
31. Xu, S. Y. *et al.* Electrically switchable Berry curvature dipole in the monolayer topological insulator WTe_2. *Nat. Phys.* **14**, 900-906 (2018).
32. Orenstein, J. *et al.* Topology and symmetry of quantum materials *via* nonlinear optical responses. *Annu. Rev. Condens. Matter Phys.* **12**, 247-272 (2021).
33. Xiao, D., Yao, W. & Niu, Q. Valley-contrasting physics in graphene: magnetic moment and topological transport. *Phys. Rev. Lett.* **99**, 236809 (2007).
34. Yao, W., Xiao, D. & Niu, Q. Valley-dependent optoelectronics from inversion symmetry breaking. *Phys. Rev. B* **77**, 235406 (2008).
35. Wu, S. *et al.* Electrical tuning of valley magnetic moment through symmetry control in bilayer MoS_2. *Nat. Phys.* **9**, 149-153 (2013).
36. Dresselhaus, G. & Dresselhaus, M. S. Fourier expansion for the electronic energy bands in silicon and germanium. *Phys. Rev.* **160**, 649-678 (1967).
37. Cheng, L. *et al.* Giant photon momentum locked THz emission in a centrosymmetric Dirac semimetal. *Sci. Adv.* **9**, eadd7856 (2023).

38. Gao, Y. *et al.* Chiral terahertz wave emission from the Weyl semimetal TaAs. *Nat. Commun.* **11**, 720 (2020).
39. Ni, Z. *et al.* Giant topological longitudinal circular photo-galvanic effect in the chiral multifold semimetal CoSi. *Nat. Commun.* **12**, 154 (2021).
40. Rees, D. *et al.* Helicity dependent photocurrents in the chiral Weyl semimetal RhSi. *Sci. Adv.* **6**, eaba0509 (2020).
41. Chen, M. *et al.* Anisotropic picosecond spin-photocurrent from Weyl semimetal WTe_2. *ACS Nano* **14**, 3539-3545 (2020).
42. Zhang, L. F. *et al.* Room-temperature electrically switchable spin-valley coupling in a van der Waals ferroelectric halide perovskite with persistent spin helix. *Nat. Photon.* **16**, 529-537 (2022).
43. Liu, X. *et al.* Circular photogalvanic spectroscopy of Rashba splitting in 2D hybrid organic-inorganic perovskite multiple quantum wells. *Nat. Commun.* **11**, 323 (2020).
44. Huang, P. J. *et al.* Chirality-dependent circular photogalvanic effect in enantiomorphic 2D organic-inorganic hybrid perovskites. *Adv. Mater.* **33**, 2008611 (2021).
45. Zhai, Y. X. *et al.* Giant Rashba splitting in 2D organic-inorganic halide perovskites measured by transient spectroscopies. *Sci. Adv.* **3**, e1700704 (2017).
46. Ishizaka, K. *et al.* Giant Rashba-type spin splitting in bulk BiTeI. *Nat. Mater.* **10**, 521-526 (2011).
47. Krempaský, J. *et al.* Disentangling bulk and surface Rashba effects in ferroelectric α-GeTe. *Phys. Rev. B* **94**, 205111 (2016).
48. Absor, M. A. U. *et al.* Defect-induced large spin-orbit splitting in monolayer $PtSe_2$. *Phys. Rev. B* **96**, 115128 (2017).
49. Kurpas, M. & Fabian, J. Intrinsic and extrinsic spin-orbit coupling and spin relaxation in monolayer $PtSe_2$. *Phys. Rev. B* **103**, 125409 (2021).
50. Zhang, Y. *et al.* Direct observation of the transition from indirect to direct bandgap in atomically thin epitaxial $MoSe_2$. *Nat. Nanotechnol.* **9**, 111-115 (2013).
51. Stroppa, A. *et al.* Tunable ferroelectric polarization and its interplay with spin–orbit coupling in tin iodide perovskites. *Nat. Commun.* **5**, 5900 (2014).
52. King, P. D. C. *et al.* Large tunable Rashba spin splitting of a two-dimensional electron gas in Bi_2Se_3. *Phys. Rev. Lett.* **107**, 096802 (2011).
53. Cho, K. S., Liang, C. T., Chen, Y. F., Tang, Y. Q. & Shen, B. Spin-dependent photocurrent induced by Rashba-type spin splitting in $Al_{0.25}Ga_{0.75}N$/GaN heterostructures. *Phys. Rev. B* **75**, 085327 (2007).
54. Caviglia, A. D. *et al.* Tunable Rashba spin-orbit interaction at oxide interfaces. *Phys. Rev. Lett.* **104**, 126803 (2010).
55. Zhang, C. *et al.* Ion-liquid-gated $KTaO_3$-based electric double layer transistor. *IEEE Electron Device Lett.* **44**, 1987-1990 (2023).
56. Nitta J. & Akazaki T, T. H., Enoki T. Gate control of spin-orbit interaction in an inverted $In_{0.53}Ga_{0.47}As/In_{0.52}Al_{0.48}As$ heterostructure. *Phys. Rev. Lett.* **78**,

1335-1338 (1997).
57. Heida, J. P., van Wees, B. J., Kuipers, J. J., Klapwijk, T. M. & Borghs, G. Spin-orbit interaction in a two-dimensional electron gas in a InAs/AlSb quantum well with gate-controlled electron density. *Phys. Rev. B* **57**, 11911-11914 (1998).
58. Luo, J., Munekata, H., Fang, F. F. & Stiles, P. J. Effects of inversion asymmetry on electron energy band structures in GaSb/InAs/GaSb quantum wells. *Phys. Rev. B* **41**, 7685-7693 (1990).
